# Taxamatch, an Algorithm for Near (‘Fuzzy’) Matching of Scientific Names in Taxonomic Databases

**DOI:** 10.1371/journal.pone.0107510

**Published:** 2014-09-23

**Authors:** Tony Rees

**Affiliations:** CSIRO Oceans and Atmosphere Flagship, Hobart, Tasmania, Australia; Consiglio Nazionale delle Ricerche (CNR), Italy

## Abstract

Misspellings of organism scientific names create barriers to optimal storage and organization of biological data, reconciliation of data stored under different spelling variants of the same name, and appropriate responses from user queries to taxonomic data systems. This study presents an analysis of the nature of the problem from first principles, reviews some available algorithmic approaches, and describes Taxamatch, an improved name matching solution for this information domain. Taxamatch employs a custom Modified Damerau-Levenshtein Distance algorithm in tandem with a phonetic algorithm, together with a rule-based approach incorporating a suite of heuristic filters, to produce improved levels of recall, precision and execution time over the existing dynamic programming algorithms *n*-grams (as bigrams and trigrams) and standard edit distance. Although entirely phonetic methods are faster than Taxamatch, they are inferior in the area of recall since many real-world errors are non-phonetic in nature. Excellent performance of Taxamatch (as recall, precision and execution time) is demonstrated against a reference database of over 465,000 genus names and 1.6 million species names, as well as against a range of error types as present at both genus and species levels in three sets of sample data for species and four for genera alone. An ancillary authority matching component is included which can be used both for misspelled names and for otherwise matching names where the associated cited authorities are not identical.

## Introduction

### The problem domain

Scientific names of organisms, together with the higher taxonomic groups within which they are nested, represent the key identifiers by which the bulk of the world's biodiversity information is organized and stored [1], yet in many cases they may be unfamiliar and non-intuitive to spell, for example *Syzygotettix boettcheri*, a grasshopper; *Cirrhitichthys oxyrhynchos*, a fish. Misspellings of hard-to-spell names, or even of more straightforward names (for example ‘Peneus’ for *Penaeus*, a common marine crustacean genus) can easily occur and, if undetected, lead to a variety of problems, including the failure to retrieve correctly spelled stored (target) data because a search (input) term is misspelled, or vice versa; stored data in one list or system not correctly matched with equivalent data in another because the spellings used are not identical; a single entered search term may not retrieve all relevant data where the latter is stored under a range of spelling variants; and in a data compilation, multiple entries for the same data item may exist under variant spellings where only one is desired, especially where the data have been aggregated from multiple sources (the ‘merge-purge’ problem [2]).

A number of taxonomic data systems currently offer some form of near match searching facility, for example utilizing the long established ‘Soundex’ phonetic algorithm [3], [4], [5]; a custom phonetic algorithm herein designated the Rees 2001/2007 near match algorithm [6], [7]; Levenshtein Distance [8]; versions of the UNIX ‘agrep’ search tool [9], [10]; and *n*-gram similarity [11], however to date, with the exception of an unpublished study by Dalcin, 2005 [12], no comparison of the relative merits of any of these approaches is available. In addition, algorithms of the dynamic programming type such as edit distance (Levenshtein Distance and related algorithms) or similarity measure (*n*-grams) require designation of relevant thresholds to be used for acceptance/rejection of particular names as candidate near matches, choice of which can significantly affect associated algorithm performance (see [12] and this study).

The present work examines the performance of selected algorithms of the above types from first principles and in practice using a range of real world misspellings of scientific names of organisms drawn from a number of sources, tested against a reference database containing over 465,000 correctly spelled genus names and 1.67 million species names, and describes the Taxamatch algorithm which is a composite approach designed with the aim of providing optimal performance for near matching of taxon scientific names. An authority comparison module is also presented which computes numeric similarities between authorities in the case that these are available for input and target names, which can either be used within Taxamatch to assist in the discrimination of likely true from false matches, or as a standalone test for measuring authority similarity in other situations.

### Near matching basics

The concept of near or inexact (‘fuzzy’) matching is well established in the wider information retrieval/computer science domain, where it may also be known as ‘approximate string matching’ or ‘string matching allowing errors’ (e.g. [13], [14]). In essence, the near matching process comprises the identification of data items (text strings) which are not identical but may be permitted to differ up to a pre-set limit, which may for example be determined by the number of characters which are different (the *edit distance* approach) or by a computed *similarity* exceeding some designated threshold according to a preferred metric. An additional requirement might be the ability to distinguish between likely true versus false hits of the same measured distance or similarity, based on characterisation of real-world errors from the domain in question, where appropriate training data is available.

Probably the most widespread application of near matching is in the numerous spell checking applications developed either as standalone programs or as integrated into a range of word processing applications offering either spell checking of pre-entered text or, in some cases, checking text as it is being typed. However, such an approach is sub-optimal for purely taxonomic data such as lists of scientific names of taxa, for the following reasons among others:

- standard reference dictionaries contain largely plain text terms (irrelevant in the present domain) and not taxonomic names; while the latter could be added, and/or plain text terms removed, the internal algorithms used in proprietary software for near matching are not generally publicly disclosed and so their performance cannot be assessed;
- scientific names of organisms have a formal internal structure (for example, species names comprise a genus name followed by a species epithet), thus an input genus name should not be tested against a species epithet, and vice versa; in addition, only epithets found in combination with a particular genus should be tested;
- the lexical characteristics of scientific names and their most common misspellings may differ from those of plain text. In other domains such as census data [15], domain-specific refinements have been introduced which substantially improve the performance of near matching algorithms according to the availability of training data, and the same should be possible for scientific names, especially if lists of real-world misspellings are available to assist algorithm development;
- algorithms for use with scientific names of organisms must be scalable so that they perform acceptably against large reference dictionaries containing (potentially) millions of correctly spelled target names, for example the estimated 1.9 million valid names for extant species [16], non-current names (synonyms) and alternative combinations for the same (perhaps an additional 100–200%, i.e. a further 2–4 million names), plus several hundred thousand names for fossil species [17] as well as those for taxa at higher ranks.

In addition, scientific names of organisms are frequently supplied with an associated authority portion which itself may require a degree of near matching, possibly using different principles from those applied to the strictly ‘scientific name’ component/s.

### The lexical nature of taxonomic names

The lexical nature of taxon scientific names offers some guidance as to how a relevant spell checker might be designed, in that (1) the names are latin in form and can possess some unusual syllables/phonemes (atomic level phonetic units) such as ‘oe’, ‘ae’ as well as leading silent characters less frequently found in plain text; (2) gender agreement of species epithets tends to produce common endings of the form -*us*, -*a*, -*um* (e.g. see [18]) which are functionally interchangeable, and thus can be allowed to be more ‘plastic’ than other portions of the word; (3) species scientific names (other than viruses) are binomial in form, comprising a genus followed by a species epithet (sometimes with an interpolated subgenus) which can define a workflow for testing these portions sequentially rather than as independent words; and (4) a variety of semi-standardized qualifiers including ‘sp.’/‘spp.’ (for unspecified single or multiple species), ‘cf.’ (comparable with…), ‘aff.’ (with affinity to…), ‘×’ or ‘x’ as hybrid prefixes, and more, may be encountered in supplied scientific name strings and may require to be catered for via a degree of pre-processing prior to actual spell checking. In additional, taxon scientific names are frequently associated with an authority (name of the scientist responsible for originally publishing the name, together with the year of publication, especially in zoology) which serves to characterise a name more uniquely than the purely ‘scientific name’ elements alone – for example *Ficus* Linnaeus, 1753 is a plant genus (the fig tree) while *Ficus* Röding, 1798 is an animal genus (marine gastropod) – and so a requirement also exists for near as well as exact matching on the authority components where these are available.

The logic of the version of Taxamatch which follows is shown for matching of genus names (uninomials) and binomial species names, with optional associated authorities, and can be extended further as desired either for higher ranks (again as uninomials), subgenera (treated as a second uninomial following genus rank), or infraspecific taxa (treated as recursive epithets) with minimal modification. To simplify matching operations for hybrids, in the hybrid genus or hybrid epithet cases (examples: ×*Agropogon* P. Fourn., *Mentha* ×*smithiana* R.A. Graham) it is simplest if the hybrid symbol is removed prior to exact or near matching operations and then added back as required following the match process, while for hybrid formulae in which the parent taxa are indicated separately (for example: *Oenothera biennis* L. × *O. villosa* Thunb.) an appropriate parsing routine should be able to separate the name elements belonging to each parent which can then be individually matched against names in the target database.

### The nature of spelling errors in plain text and in the ‘scientific name’ portions of taxonomic names

Kukich [19] classifies spelling errors into typographic errors, cognitive errors and phonetic errors, where typographic errors arise from motor coordination slips such as hitting an incorrect key, or inadvertent omission, insertion, or reversal of typed characters; cognitive errors result from a mis-apprehension of how a word should be spelled; and phonetic errors are a subset of the previous class in which similar-sounding but incorrect syllables or phonemes are substituted for correct ones. To this list can be added other types of error, including for example inadvertent truncation (e.g. the final characters do not fit in a supplied text entry box, or break over two lines in published text); optical character recognition (OCR) errors, such as mistaking *m* for *rn*, *f* for *t*, together with a similar class of errors arising from transcription from handwriting; transmission/encoding errors, such as those which arise due to incorrect character encodings, for example non-ASCII characters such as those with diacritical marks can frequently be represented differently in different computer systems; and probably more.

In the context of taxon scientific names, such errors manifest themselves primarily as either phonetic (soundalike) errors, for example *Peneus* for *Penaeus*, *forti* for *fortii*, or non-phonetic (e.g. mis-keying) errors such as *Acropaginula* for *Arcopaginula*, *flaveolata* for *faveolata*, and so on. The special case of gender-specific endings for species epithets has been mentioned above, with the consequence that, for example, *Neocrex columbiana* and *Neocrex columbianus* can be treated as variant forms of the same species name (the former correct, the latter incorrect, refer [20]) and should therefore be accommodated as such by a designated ‘taxonomic’ near match routine.

With regard to the potential magnitude of spelling errors, Damerau [21] contended that around 80% of errors in his sample of plain text misspellings affected a single character only (including reversals) while the remaining 20% affected more than one character and so should not be ignored for satisfactory error correction performance. Kukich [19] mentions the belief that errors infrequently affect the leading character of a word but points to published studies in which the occurrence of such errors varies from 1.4% to 15%, so the correct assignment of the leading character cannot be automatically assumed (as it is, for example, in the Soundex algorithm) without losing recall. Another potential influence is that of word length, in that, for example, a 3-character error might be tolerated in (say) a 10-character word but not in a 3-character word (or all such words would automatically match each other, a clearly nonsensical result). There do not appear to be previous studies addressing this issue in particular, however it proves to be a useful consideration for the design rules of Taxamatch as will be outlined below.

Algorithms to address the two main classes of errors introduced above (phonetic versus non-phonetic) differ substantially in their design and associated performance aspects. ‘Phonetic’ algorithms rely in the main on the construction of a phonetic representation or ‘key’ as a transformation of both input and available target terms, and if the two keys match (but the terms are not identical) then a phonetic match is reported. As an example, the character ‘h’ is frequently considered to be silent and might be dropped, and ‘i’ and ‘y’ may be considered phonetic equivalents, thus a phonetic key for both the surnames ‘White’ and ‘Whyte’ might be the same, e.g. represented as ‘WITE’ after transformation to uppercase (to reduce mismatches due to case alone). This type of near match lookup can be made very efficient ( = rapid) at search time by the simple expedient of computing the phonetic keys for all target terms in advance, leaving only the transformation of the input term to be done at run time, followed by an exact match comparison on the phonetic keys: this can operate very quickly, being a standard data operation, especially in a database environment with the facility to pre-index all terms in a separate portion of the database structure for enhanced performance at search time (for example refer [22]).

By contrast, non-phonetic algorithms rely almost exclusively on some form of live dynamic programming activity (a series of calculations to be executed at run time) since the exact relationship between the two terms to be compared cannot usually be modelled in advance. For example, implementation of the popular Levenshtein Distance measure of edit distance involves the creation and traversal of a 2-dimensional array of characters in order to calculate the required distance [23], [24] which, although quite fast for a single pairwise comparison, becomes slow to execute against a set of thousands or potentially millions of stored target names, especially in comparison to the near-instantaneous result obtainable using a simple test to compare phonetic keys. This, then, creates a problem for a comprehensive spell checking solution which seeks to detect non-phonetic as well as phonetic errors, since ideally we would like a result returned to a user without undue delay (for example within seconds rather than minutes or hours, even against a large reference dataset), and in addition it is desirable to minimize the time it takes to deduplicate a large system which involves testing all names against each other (for example in a data system containing 2 million names this would potentially require 4 billion pairwise comparisons). Addressing the issue of algorithm execution time then becomes a key consideration for the design of Taxamatch, as will be explored further below.

### Mismatches in the authority portions of taxonomic names

As introduced above, near matching on the authority portion of a taxonomic name may also be a design goal, which then requires some exploration of the lexical characteristics of such authorities and areas in which cited versions of essentially the same authority can differ. Such differences may include abbreviation of an author surname (as is common in botany), for example ‘L.’ versus ‘Linnaeus’; addition or omission of a publication year, for example ‘Röding’ vs. ‘Röding, 1798’; variation in citation format for multiple authors, for example ‘Rees et al., 1974’ vs. ‘Rees, Leedale & Cmiech, 1974’; inclusion or omission of author initials and/or suffixes, such as ‘Loeblich’ vs. ‘A.R. Loeblich’ vs. ‘A.R. Loeblich, Jr.’; variation in citing author teams with the same surname, such as ‘H. & A. Adams’ vs. ‘H. Adams & A. Adams’; variability in citation of a name ascribed to an author other than that of the containing publication, for example ‘Prins, 1974’ vs. ‘Prins in Grün et al., 1974’; variation in supplied diacritical marks, for example ‘Müller’ vs. ‘Mueller’ vs. ‘Muller’, and more, in addition to simple misspellings of author names analogous to those which may be encountered in the scientific name portions. Such potential differences are clearly much less straightforward than could be reasonably treated by either a phonetic or an edit distance approach as will be discussed for scientific names, and will accordingly be approached via a different route (in fact, combination of routes), as explained further below in step 8 of the Taxamatch algorithm description section.

### Selected algorithms and metrics for comparing text strings

An overview of some commonly used phonetic algorithms in the context of personal name matching is given in Christen [25] including descriptions of Soundex, Phonex, Phonix, NYSIIS (New York State Identification Intelligence System) and more. The Soundex phonetic algorithm (and some of its derivatives) is, however, fairly non-selective in two respects: first, a comparatively large range of characters are equated for matching purposes (for example in Soundex, all vowels are considered equivalent, as are groups of somewhat similar sounding consonants such as c, g, j, k and q), and second, the phonetic keys typically represent only the leading 4–6 characters and the remainder of the word is commonly ignored. For taxonomic names, neither of these characteristics are considered to be ideal (with the potential to lead to an elevated number of false positives) and so a custom algorithm was created in 2001, further refined in 2007 (the ‘Rees 2001’ and the ‘Rees 2007’ phonetic algorithms described herein) which undertakes a more controlled subset of phonetic substitutions and in addition treats the entire word independent of length (the Rees 2007 version also incorporating the gender normalization rules for species epithets described above, which although not strictly phonetic substitutions, can be implemented in an exactly analogous manner). The Rees 2007 phonetic algorithm is also deployed for specific tasks within Taxamatch, both to assist some rapid pre-processing at both genus and species level, and to report phonetic matches separately from non-phonetic ones according to the principle that these may be more plausible where present. It should be further noted that phonetic algorithms such as those considered in this work are essentially Boolean (binary) in operation: either two text strings are designated as a phonetic match or they are not, giving a simple pass/fail result for tests of this type, in contrast to the dynamic algorithms where varying degrees of distance or similarity can be reported and used for specific ranking and dynamic filtering purposes as desired.

Non-phonetic algorithms typically measure either *distance* (on a scale 0-*x*, where 0  =  identical and increasing values of *x* indicating increasing dissimilarity) or *similarity*, typically reported on a 1–0 scale where 1  =  identical and 0  =  no similarity. Again, a good summary of available metrics is found in [25] including the distance measures Levenshtein or Edit Distance, Damerau-Levenshtein Distance, Bag Distance and Smith-Waterman Distance, with similarity measures represented by *n*-grams (designated therein as *q*-grams), positional *q*-grams, skip-grams, normalised compression distance (NCD), plus the Jaro, Winkler, sorted-Winkler and permuted-Winkler similarity measures, while other authors e.g. Cohen et al. [26] present yet more options including Jaccard similarity, cosine similarity, Jensen-Shannon Distance, Monge-Elkan similarity and more. Taking first the distance measures, the simplest (Levenshtein Distance, after Levenshtein [30], hereinafter abbreviated LD) measures single character insertions, deletions and substitutions, each contributing a distance value of 1 to a cumulative count: therefore the Levenshtein Distance between *Fucus* and *Ficus* = 1 on account of the single character substitution (of ‘i’ for ‘u’) being all that is required to transform the first string into the second. LD however does not account for transpositions (reversals), thus the edit distance calculated as LD between ‘aslo’ and ‘also’ is 2 (two independent substitutions), not 1 as might be preferred considering that this can be better regarded as an error resulting from a single motor coordination slip. For this purpose an extension of the LD test has been introduced, first by Lowrance & Wagner [27] and christened by subsequent workers the Damerau-Levenshtein Distance (herein: DLD), which also recognises transpositions as single character operations and accords them a weight of 1, the same as a single insertion, deletion and substitution.

In the course of the present work, an analogous case was encountered on a small number of occasions where the transposition affects not one but two adjacent characters, for example *Panulirus* vs. *Palinurus*, *serratulus* vs. *serrulatus*, each of which examples would be accorded an undesirably large edit distance (ED) of 4 under either the LD or DLD measure. To compensate for this effect, an extension of DLD has been developed for this work which is termed Modified Damerau-Levenshtein Distance or MDLD, which recognises the case(s) of multiple character transpositions (for example transposed 2- or 3-character blocks) and accords them the weight of the length of the transposed substring rather than double this number which would be the case if all changes were treated as independent substitutions. This MDLD measure is then placed at the core of the Taxamatch algorithm where it is employed for the edit distance calculation at both genus and species epithet levels.

Edit distance tests such as the above have been preferred for Taxamatch comparisons involving the scientific name components since errors in these elements appear to closely follow the principle of a relatively small number of character insertions, deletions, substitutions or transpositions as previously documented by Damerau [21] for plain text and Dalcin [12] for taxonomic names. By contrast, mismatches in supplied authorities have the potential to involve much more than just a few isolated characters and for this purpose any usable threshold of edit distance is likely to be seriously inadequate, thus a measure which detects residual similarities rather than differences is considered to be more appropriate in this case. For this reason, an approach has been constructed using the relatively well established similarity measure *n*-grams which are short, overlapping substrings of both input and target terms of length *n* characters, commonly used values of *n* being 2 or 3, the *n*-gram similarity being computed as the ratio of common *n*-grams between the terms to the mean number of *n*-grams in each term. As an example, for *n* = 2 (bigrams), the word ‘Tony’ (converted for example into uppercase) contains the *n*-grams ‘TO’, ‘ON’ and ‘NY’ while ‘Toby’ contains ‘TO’, ‘OB’ and ‘BY’, a similarity measure of 1 out of a possible 3 or 0.333. However, closer inspection of this initial approach reveals that the terminal characters ‘T’ and ‘Y’ are under-represented, appearing in only one *n*-gram each, as opposed to the internal characters which occur in multiple *n*-grams, therefore to remove this discrepancy it has been suggested to pad both terms to be compared with *n*−1 blank characters prior to substring generation (refer [28] and elsewhere). After such padding, with a blank character indicated by * the string ‘Tony’ now has the bigrams ‘*T’, ‘TO’, ‘ON’, ‘NY’ and ‘Y*’ while ‘Toby’ has the bigrams ‘*T’, ‘TO’, ‘OB’, ‘BY’ and ‘Y*’ giving arguably a more ‘realistic’ calculated similarity of 3/5 or 0.6.

In contrast to edit distance measures, it will be apparent that calculated similarities using *n*-grams or analogous measures are typically word length dependent, in that a single character error in a short word will tend to affect the calculated similarity more than the same error in a longer word. *N*-grams will also be more affected than edit distance measures (at least in the case of LD and DLD) by transposed characters, since these will affect more adjacent *n*-grams than simple substitutions, insertions and deletions. Interestingly, bigrams turn out to be unaffected by word order, in that the calculated bigram similarity between ‘Smith & Jones’ and ‘Jones & Smith’ is 1 since they contain exactly the same bigrams once padding is introduced as discussed above (this is not the case for padded trigrams, which return a similarity value of 0.7333 for the same pair of terms). Accordingly, the choice of which *n*-gram method(s) to employ will have some implications when it comes to incorporation into relevant comparison routines. (Additional similarity-based measures have not been considered at this time since, in the present use case which is for authority comparisons, *n*-grams appear to function adequately to indicate a first-order similarity when two strings have at least some character patterns in common).

One final comment which applies to the dynamic tests (edit distance and *n*-gram measures in the present context) is that, in addition to their overall slower execution time in practice as discussed above, such tests will in general scale non-linearly in execution time according to the mean length of input and target terms: for example a set of 20×20 characters (example binomial length) will in principle require four times as long to compare as a set of 10×10 characters (example uninomial length) on account of requiring four times the number of individual character-by-character comparisons. Therefore, as will be shown in the next section, an approach which tests the genus and species epithet portions of a binomial name separately could potentially have a 2 times speed advantage over testing the entire name as a single string.

### Algorithm metrics and optimization potential

#### Maximizing recall: retrieving ‘all’ true matches

**Figure 1:**
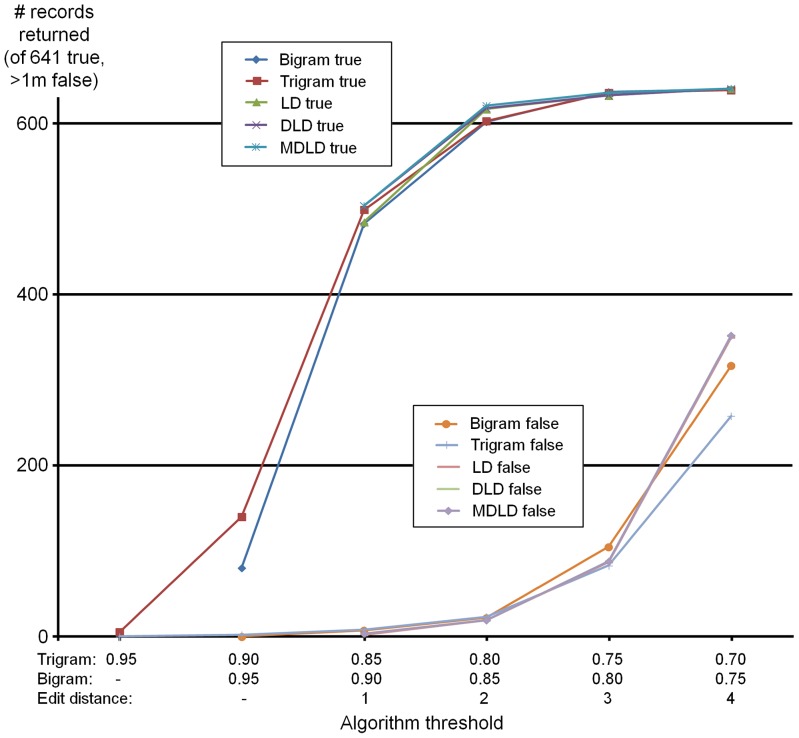
Sample species-level algorithm performance (as true vs. false hits returned) with increasing threshold settings. Performance of five dynamic algorithm variants using the CAAB expert misspellings species dataset at a range of thresholds. Horizontal alignment and scales are chosen to emphasize general similarities in response patterns between otherwise different algorithms. Curves for LD and DLD lie under that for MDLD where not visible.

Within the general domain of information retrieval, algorithm performance is commonly assessed against three leading criteria: recall, precision (these also being combined as *effectiveness*) and efficiency [29], [30]. *Recall* is defined as the proportion of ‘relevant’ documents retrieved by a given query out of the number actually present in the target data storage system, usually on a 0–1 scale. In the present context the result in individual cases will by definition be either 1 or 0, therefore to get a representative metric we will calculate the value averaged over all queries in a given set, calculated as a fraction of 1 where 1 indicates perfect recall (correct target returned on 100% of occasions) and 0 indicates that correct targets were never returned. In the case of the dynamic algorithms where thresholds can be varied at will, it is of course possible to obtain a recall value of 1 by simply setting relevant thresholds to exceed the maximum distance (or minimum similarity) present in the data. Such behaviour is represented in Figure 1 which includes a representation of recall (as number of true hits in this instance) in the dataset of misspelled species names used as training data (further described in Materials and Methods): in this case an edit distance of 4 (as either LD, DLD or MDLD) is sufficient to return all designated ‘correct’ targets (at genus level the equivalent value is 3), while for bigrams a similarity threshold of 0.688 is sufficient to return all designated target species, and 0.667 for genera alone (for trigrams the equivalent values are 0.516 for species and 0.609 for genera). However, along with such wide or relaxed thresholds, the contribution of false hits can become enhanced as also visible from Figure 1, which therefore requires a strategy to manage as described below.

#### Maximizing precision (minimizing false hits)

*Precision* is defined as the proportion of ‘true’ to ‘all’ hits present in the returned near match set, which may conceivably contain a percentage of false positives in addition to true hits, so the closer this value can be made to 1 on a 0–1 scale, the better. Inspection of Figure 1 indicates that false hits can be suppressed reasonably effectively at narrower thresholds such as ED 1 and 2 (or similarity thresholds of e.g. 0.8 and above), however performance degrades at larger values of edit distance (or reducing similarity thresholds for the *n*-gram based measures). As an operational strategy, one option here might therefore to be to mimic what a human operator might do faced with a potentially misspelled name: first look for lexically close hits using a narrow threshold, and only widen this in the case that no result is obtained. Such an approach is incorporated into Taxamatch where it is designated *result shaping* (in essence a type of dynamic filter), although as will be explored further below there may be situations where this results in some more distant desired true hits being suppressed and, for that reason, it is suggested to make this facility a switchable option for disabling in some circumstances only.

A second approach, also incorporated into Taxamatch, is to attempt to reduce the false positives associated with any particular threshold setting via additional filtering based on a suite of heuristic rules designed to match the lexical characteristics in real world sample data (see [31], [32], [33]) but in this case from the domain of misspelled taxonomic names. To take a well known example, one such rule, included in the Soundex and the Rees 2001 phonetic algorithms, is to require that the leading character of input and ‘approved’ target terms must always match. However, inspection of sample data indicates that this condition is not always fulfilled. One might therefore experiment with modifying the initial rule and mandate that where the leading character is different, the final character (or multiple characters) must be the same, and re-test using the sample data; this process is then continued iteratively until a rule (or set of rules) is devised which appears to suppress the maximum practicable proportion of false hits without impacting negatively upon recall of true hits.

Application of this approach then leads to the creation of *post-filters* following the edit distance test at both genus and species epithet levels, which incorporate the relevant heuristic rules and serve to enhance algorithm precision in each case. In fact, these rules can be further divided into those which require the measured edit distance to be known and those which do not: for example, if a rule is based on observed lexical patterns such as substring matching (possibly in conjunction with word length) independent of measured edit distance, then term pairs which fail this rule need never be tested by the computationally expensive dynamic term comparisons but rejected at an earlier stage, i.e. incorporated into relevant *pre-filters* as described in the next section.

#### Optimizing algorithm efficiency (reducing execution time)

In the domain of taxonomic names, reference target compilations can be very large, for example at time of writing, The Plant List [34] contains over 1 million names, while the Catalogue of Life [35] and the Global Names Index [36] contain over 2 million and 17 million names, respectively. However, dynamic processing algorithms are essentially slow: for example, the MDLD comparison takes around 0.5 milliseconds per name pair (this study) and on that basis would require 500 seconds (more than 8 minutes) to test a single input name against all targets in a reference database of 1 million names, which is clearly undesirable for live user queries in particular. It is therefore essential to consider ways by which overall execution times per input query can be reduced to an acceptable level (perhaps a second or two at maximum), especially against large reference datasets. Potential strategies to achieve shorter execution times include:

- selection of the fastest algorithm which possesses acceptable precision:recall characteristics for the desired task;
- splitting binomial names into separate portions for testing, which, for dynamic algorithms as mentioned above, will typically lead to an improvement in individual processing time even if the two tests are then summed;
- reducing the number of individual name pair tests to be carried out, ideally without eliminating any desired true hits in the process.

The first of these is revisited later in the section on ‘further improving Taxamatch execution time’ but in the present context it is preferred to use the algorithm which performs best against the full range of error types encountered in real world misspellings of taxonomic names, the MDLD test, as will be described further in the experimental section. The second strategy is incorporated into Taxamatch in that genus and species epithets are tested individually, rather than as a combined text string. This then also feeds into the third option which is to reduce the number of tests to be carried out: if for example the ratio of species names held to genera is in the order of 10∶1 (in practice this might vary from around 4∶1 to 60∶1 but the principle still holds), then testing the genera first will reduce the number of initial tests considerably (e.g. by up to 90%) and then the only species epithets to be tested would be those associated with candidate ‘near match’ genera, a further significant saving. In addition to the constraint just mentioned, the set of names to be tested can be reduced still more by the addition of pre-filters at both genus and species epithet levels, based on logical principles. For example, if a maximum edit distance of 3 is to be permitted in the genus match test, then it is unnecessary to test target genus names greater than 3 characters shorter or longer than the input genus since their edit distance can never be within the permitted threshold (a minimum of 4 insertions or deletions will always be required to make the two terms match); for binomials the maximum permitted threshold is 4 so target epithets more than 4 characters shorter or longer need not be tested. (In practice, it is found that an even tighter threshold of 2 can be employed in the genus pre-filter, provided that phonetic matching is also permitted as an alternative which can bypass this threshold as required). If we then add into the same pre-filters, those heuristic filtering rules previously created which do not require completion of the edit distance test, an even higher degree of selectivity can potentially be achieved.

We can test the possible efficacy of such an approach by inserting some hypothetical values into a sample workflow: let us suppose a reference database contains 1 million species names associated with 1,000 genera, of which only 100 genus names pass the relevant genus pre-filter (a 90% selectivity) and are tested against a given input name, and of these, 50 of these pass a genus post-filtering stage and the remainder are rejected. These 50 ‘candidate near match’ genus names have perhaps 500 species epithets associated with them of which 50% are rejected by a species pre-filter, leaving 250 to be tested before the overall result set is returned. In all we have then tested only 100 genus names and 250 epithets, a total of 350 tests as opposed to the original 1 million species names which would be tested in the naïve/brute force case, a saving of over 99.96% (some comparable real test data are supplied later in this study). Furthermore, the individual tests are carried out on shorter text strings (either a genus or species epithet alone) than on the full binomial, an additional potential 50% time saving owing to the quadratic penalty associated with increasing word length.

### Additional components for a total solution

Combining the elements above produces the core of a workflow for comparing taxonomic names, but some additional elements are still required, as follows:

- an initial pre-processing step of *name parsing* and *normalization* may be necessary (unless the name elements are already atomized and normalized). Name parsing in this context means analysing the supplied input term and atomising the relevant portions corresponding to genus, specific epithet (where supplied) and authority (also where supplied) for separate treatment within the algorithm workflow, also allowing for the possible presence of rank indicators, subgenera in parentheses, and any stray text which is not strictly a portion of the taxonomic name.
- an *authority comparison* stage is provided, for reasons stated above. In the present suggested workflow this is applied once the set of near match scientific names has been generated in order to avoid undue computation, and can be implemented at either genus or species level according to the rank for which the authority is supplied, although as desired it might be applied to a wider set of names for other purposes;
- a *ranking* step is typically useful, for example it may be desired to rank the results by edit distance in the case that multiple near matches are available, and/or present phonetic matches separately from the others. As an alternative, it may be desired to present hits with the best authority match as the leading candidate, where such information is available for both input and target terms.

**Figure 2:**
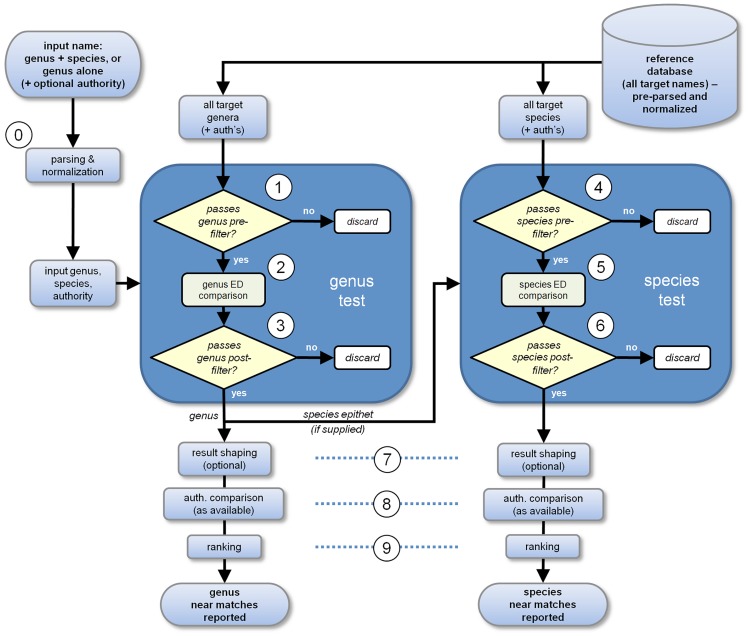
Overall schematic of an optimized algorithm for comparing taxonomic names as described above and implemented in Taxamatch.

Combining all the elements described above it is possible to produce an ‘ideal’ algorithm workflow which is presented in Figure 2, and is used for the design of Taxamatch; details of operations presented as numbered steps 0 to 9 are described in the next section. For simplicity, only the available target genus names are shown as an input to the genus test although in practice, some reference to their associated species is also required as an input to the genus pre-filter. The main dynamic programming comparison of both genera and species is undertaken using the relatively expensive MDLD test but the cost of this is mitigated considerably by the operation of the genus and species pre-filters as described in the previous section.

## Taxamatch Algorithm Description

The main Taxamatch algorithm is presented below. The pre-processing (parsing and normalization) stage is presented as an (optional) ‘step 0’, since this may not always be required as a part of normal Taxamatch operation: for example in the case of internal deduplication or comparison of already well structured data such steps will potentially already have been applied, and in the author's system they are incorporated in all cases as a precursor to exact matching and need not be repeated for the near match. Additionally, it is possible that name parsing might not be undertaken within the Taxamatch workflow but by making use of a separate module such as the Global Names Parser available from [37].

### Step 0: Pre-processing (name parsing and normalization)

As introduced above, the requirement for parsing is to identify and then isolate the required name components for treatment by relevant portions of the algorithm. As an example, from the input name ‘*Fagus sylvatica* L.’ we would like to extract genus  =  Fagus, species epithet  =  sylvatica, species authority  =  L. while from ‘*Fagus sylvatica* subsp. *orientalis* (Lipsky) Greuter’ (example given in [37]) we would like genus  =  Fagus, species epithet  =  sylvatica, subspecies  =  orientalis and subspecies authority  =  ‘(Lipsky) Greuter’ (if matching were continued to subspecies level). Other parsing actions which might be required include identifying subgenus names where supplied (for example in parentheses between a supplied genus and species epithet); identifying and possibly removing qualifier (uncertainty) terms such as ‘cf.’, ‘aff.’ and ‘?’; stripping and/or replacement of stray HTML mark-up such as ‘<i>’, ‘</i>’, ‘ ’ (the latter to be replaced with an ampersand); and dealing correctly with hybrid symbols and hybrid formulae, as discussed above. A degree of normalization is also desirable which might include transforming all scientific names to uppercase, removal of hyphens and leading or trailing white space, and replacing any accented characters or ligatures with their plain ASCII equivalents (normalizing of authorities ideally has slightly different requirements and is dealt here separately under step 8).

Whatever parsing and normalization routines are used, it is of course required that they be applied consistently to both input and target terms, and also that operations on the latter are preferably carried out in advance (with suitable indexing applied) in order to avoid undue performance penalties at query time.

### Step 1: Genus pre-filter

For this step it is presumed that the target set of unique genus names can be queried either as a real set (for example in a ‘genus’ table) or as a virtual set, i.e. the set of unique genus names forming portions of species names in a list; in either case the intention is to avoid testing the same genus name more than once, even though it may be associated with multiple species. This step comprises 3 heuristic rules developed from training data, satisfying any one of which is sufficient to pass the name to the next step for testing:

- EITHER: (*Rule 1a*) The genus portion of the input name and the genus portion of the target name are a phonetic match, as indicated by the ‘Rees 2007 near match’ algorithm described herein;
- OR: (*Rule 1b*) The species epithet portion of the input name and the species epithet portion of the target name are a phonetic match (including allowance for possible gender variation), AND the difference in (normalized) length of the input and target genus names does not exceed 3 characters;
- OR: (*Rule 1c*) The difference in length of the input genus and target name does not exceed 2 characters AND selected substrings of the input genus name and target genus match (variable according to word length as below), as follows:
  1. Minimum genus length (i.e., shorter of input and target names) <5 characters: require a match on EITHER the first character OR the last character only;
  2. Minimum genus length 5 characters: require a match on EITHER the first 2 OR the last 3 characters;
  3. Minimum genus length 6 characters and above: a match on EITHER the first 3 OR the last 3 characters.

### Step 2: Genus comparison

This uses the MDLD algorithm (refer File S2 for an implementation in Oracle PL/SQL) to return the edit distance (ED) between the input genus and every target genus name passing the genus pre-filter. In addition, if either the genus or species names are a phonetic match as determined in step 1, the latter information is carried through to subsequent steps to be used as required. In principle the MDLD algorithm can test for transposed character blocks of any length; in practice, setting the maximum allowable transposed block length to 2 characters returns all true hits (with sample data) while permitting a shorter average execution time.

### Step 3: Genus post-filter

This comprises three heuristic rules as follows, again designed using training data:

- *Rule 3a*: Genera with ED of 4 or above are discarded;
- *Rule 3b*: Remaining genera which are a phonetic match (on the basis of the Rees 2007 phonetic algorithm) are automatically accepted;
- *Rule 3c*: For remaining (i.e. non phonetic match) genera at least 50% ‘good’ characters are required (i.e. a maximum of ED 1 is permitted in a 2 or 3 character word, maximum ED 2 in a 4 or 5 character word, maximum ED 3 in a 6+ character word), AND the initial character must match in all cases of ED 2 and above (other genera are discarded).

Note that Rule 3c is a compromise between the requirement for leading characters to match in all cases (which is considered to be too strict, refer present work and also [38]) and ignoring this requirement altogether (which leads to an undue proportion of false hits being included in the relevant result set). Rule 3b which accepts phonetic matches will also allow through a subset of names which do not strictly match on the leading character but which sound equivalent, such as potential substitution of ‘PH’ by ‘F’, etc., at the word start.

At this point the genus portion of the input name (which may be the only portion if no epithet is supplied) can, as desired, proceed to result shaping, authority comparison if available, and ranking which are described below in steps 7–9, while in the case of binomial names, the species epithet is additionally passed to step 4.

### Step 4: Species pre-filter

This comprises a single additional, length-based constraint (rule 4b) in combination with the hierarchical relation with genus names as passed from step 3 (rule 4a):

- *Rule 4a*: Target species must be a child of one of the set of genera passing the genus post-filter;
- AND *Rule 4b*: The difference in length of the input species epithet and target epithet does not exceed 4 characters.

### Step 5: Species comparison

This uses the MDLD algorithm to return the edit distance (ED) between the input species epithet and every target epithet passing the species pre-filter. In this case a maximum block limit of 4 transposed characters can be allowed since the number of epithets to be tested by this stage as relatively small, as discussed above, and execution time is not normally a limitation.

### Step 6: Species post-filter

The following heuristic rules are applied in this step:

- *Rule 6a*: The combined edit distance of genus and species epithet must not exceed 4 (others are discarded);
- *Rule 6b*: Remaining epithets which are a phonetic match are automatically accepted;
- *Rule 6c*: For remaining (i.e. non phonetic match) epithets at least 50% ‘good’ characters are required (i.e. a maximum of ED 1 is permitted in a 2 or 3 character word, maximum ED 2 in a 4 or 5 character word, maximum ED 3 in a 6 or 7 character word, maximum ED 4 in a 8+ character word), AND:
  1. for all cases of [species epithet] ED 2 and 3 the initial character must match;
  2. for all cases of [species epithet] ED 4 the initial 3 characters must match.

### Step 7: Result shaping

The basic principle of the result shaping step of Taxamatch is in essence, to return ‘close’ near matches where present without requiring to widen the threshold unduly (thereby reducing the return of false hits in most cases), without losing the facility to detect more distant hits in the case where closer ones are absent. When result shaping is not required (the Taxamatch ‘no shaping’ mode) all near matches are reported and this step is omitted. Otherwise with result shaping enabled, the following rules are employed, with minor differences according to whether a genus name or a species epithet is being treated:

- *Rule 7a*: Names at ED 0 are returned as exact matches (alternatively they may already been identified via a separate ‘exact match’ pass, in which case they can be excluded from consideration by Taxamatch at an earlier stage);
- *Rule 7b*:
  1. For species, names at ED 1 + ED 2 (including errors in both genus and species epithet) plus phonetic matches are always returned, where present;
  2. For genera, names at ED 1 plus any phonetic matches are always returned, where present;
- *Rule 7c*:
  1. For species, names at ED 3 are only returned in the absence of ED 1, ED 2 or phonetic matches;
  2. For genera, names at ED 2 are only returned in the absence of ED 1 or phonetic matches;
- *Rule 7d*:
  1. For species, names at ED 4 are only returned in the absence of ED 1, ED 2, ED 3 or phonetic matches;
  2. For genera, names at ED 3 are only returned in the absence of ED 1, ED 2 or phonetic matches.

### Step 8: Authority matching

8.1 The following authority-specific normalization steps are recommended as pre-processing:

- normalize ‘et’ and ‘and’ to ampersand character (except in the special case ‘et al.’ which is retained unchanged), also any HTML equivalent i.e. ‘&’ becomes an ampersand;
- normalize presentation of white space after full stops in the case of author initials (e.g. ‘F. J. R. Taylor’ becomes ‘F.J.R. Taylor’, or vice versa);
- normalize presentation of commas before dates (e.g. ‘Taylor 1971’ becomes ‘Taylor, 1971’, or vice versa);
- expand any apparent abbreviated author surnames detected (if supplied) using a stored dictionary of known author abbreviations and their expanded forms (for relevant detail refer Implementation section);
- normalize the strings to uppercase for comparisons.

8.2 If one string to be compared ends with a date (with or without a final bracket) and the other does not, remove the last 3 digits from the date component in the string which contains this prior to comparison, to reduce but not entirely eliminate the influence of this discrepancy, such that, e.g. ‘Linnaeus, 1758’ becomes ‘Linnaeus, 1’; ‘(Linnaeus, 1758)’ becomes ‘(Linnaeus, 1)’ (the contribution of the retained single number plus preceding space and comma indicate that there is some residual difference between the strings, but does not overwhelmingly affect short authority strings).

8.3 If one string commences with a bracket and the other does not, remove the leading bracket in this case prior to comparison, e.g. ‘(Linnaeus 1758)’ becomes ‘Linnaeus 1758)’ (rationale: reduces by 50%, but does not entirely remove the effect of this discrepancy).

8.4 Create a second (‘plain’) version of each string, with characters employing diacritical marks and ligatures replaced by their plain ASCII equivalents (‘Lacépède’ becomes ‘Lacepede’, ‘Sæther’ becomes ‘Saether’, etc.).

8.5 The authority *n*-gram comparison is now calculated using a weighted blend of padded bigrams (*n* = 2) and padded trigrams (*n* = 3) (bigram similarity is generally preferable for shorter words but, as previously noted, this has the non-optimal characteristic of being insensitive to word order; for that reason, a blend of 2/3 bigrams with 1/3 trigrams is utilized, so that the impact of variation in word order is reduced but not completely eliminated). This process is undertaken twice, once for the original string (with accented characters retained if these exist) and once for the ‘plain’ form with diacritical marks removed, then the mean of the two values is returned (note that if no accented characters are present, i.e. original and ‘plain’ forms are identical, this calculation need only be carried out once).

### Step 9: Ranking

As discussed earlier, this is to some degree a matter of designer preference. In the author's system the following rules are applied:

- *Rule 9a*: candidate near matches at ED 1 plus any phonetic matches are returned as ‘nearest matches’ at both genus and species levels (alternatively, phonetic matches could be singled out for special designation as the most plausible set);
- *Rule 9b*: remaining near matches are returned as ‘other near matches’ for separate consideration (since they may also contain a true hit on occasion, but less frequently than the ‘nearest match’ set).

In the author's reference system authority similarity is not used as a ranking criterion since on occasion it may give a misleading result (for example when the same author name is cited in very different ways), but is included in result presentation as a visual aid since in most cases it is helpful to the human user, when available.

A pseudocode representation of the entire Taxamatch algorithm is presented as File S3, in conjunction with File S2 which contains Oracle PL/SQL implementations of the MDLD algorithm for reference (the ‘Rees 2007 near match’ and *n*-gram algorithms can be created from first principles following the information presented in Materials and Methods, or downloaded from the Taxamatch web site [39]).

### Implementation aspects

**Figure 3:**
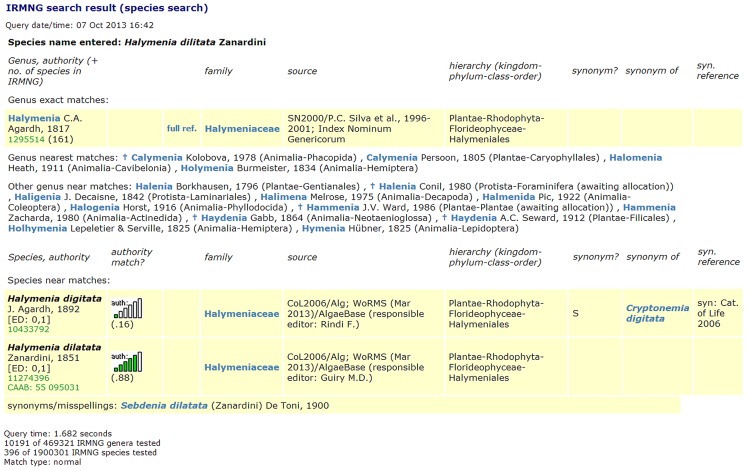
Example search result from the author's Taxamatch-enabled IRMNG data search as at May 2013. Sample result screen from Taxamatch-enabled search via the current (2013) implementation of the IRMNG database web search interface using as input the misspelled name *Halymenia dilitata* Zanardini, an error for *Halymenia dilatata* (genus exact match, ED 1 near match in species epithet). Note the additional return in this case of the false hit *Halymenia digitata* J. Agardh at the same edit distance, however a poorer match on authority (0.17 vs. 0.88; species ordering is alphabetic), also the return of multiple near match genera (even if no near match species is currently held in those genera) since on occasion the desired target species may be missing from the reference database but the target genus may not.

For Taxamatch implementation in an operational system, the following components are required:

- routines for parsing of input text strings if required (in the event that text is not already pre-supplied as e.g. genus, species epithet and authority portions) and then normalization as described above in steps 0 and 8 (separately for scientific name and authority portions);
- programmatic implementations of the following:
  - the ‘Rees 2007 phonetic algorithm’;
  - the Modified Damerau-Levenshtein Distance (MDLD) edit distance test, and
  - if authority comparisons are desired, a facility to calculate padded *n*-gram similarities between supplied strings for both bigrams and trigrams;
- a reference set of notionally correctly spelled target names (with authorities as preferred) against which input names can be tested (if it is desired to further offer the facility to restrict testing to a particular taxonomically or otherwise circumscribed subset as will be discussed later, equivalent necessary indicators or flags will also need to be available in the reference database);
  Note, in order to speed Taxamatch operation, it is recommended that additional derived columns (with appropriate indexes) are created in this reference database and also it will be necessary to update such columns in the event that relevant content is changed or new rows added. For genus level data the desirable additional columns applicable to each stored name are genus length (in characters), normalized genus name, and ‘Rees 2007 near match’ transformed version of the genus name, while for species, equivalent columns are required, for the species epithet only, together with a pointer to the containing genus name in the event that this is held in a separate table (as is recommended for operational efficiency.
- a reference set of known author abbreviations together with their full (expanded) equivalents, in the case that the authority comparison module is implemented;
- a means for users to submit either single input names or lists of names for testing, as desired, plus relevant methods to return candidate near matches to the user (e.g. as a web page or supplied report); alternatively, for internal deduplication or list matching purposes, custom code that calls Taxamatch on a recursive (i.e. row-by-row) basis and deals appropriately with the results (for example writes to relevant additional database columns, or generates a report of some kind);
- a sufficiently well specified computing platform to provide acceptable response times to real time user queries and if desired, larger internal deduplication tasks, according to target database size (an example specification for the author's present reference system is detailed in Materials and Methods); and
- desired presentation and formatting choice/s for what information is to be returned in response to user input. In the author's reference Taxamatch implementation operating over the IRMNG (Interim Register of Marine and Nonmarine Genera) database at CSIRO [40], in addition to the ranking measures described in step 9 above, the edit distance is reported for every match using the format ‘x,y’ for genus and species epithet portions, respectively, and the calculated authority similarity is reported where this is available, as both a value and as an associated small graphic (bar chart with filled versus unfilled columns) to facilitate rapid visual assessment. Supplementary information regarding the taxonomic and other status of each returned name is also shown, obtained via queries to relevant columns and other tables once the sets of near match names have been constructed. In addition (for ongoing system performance assessment) the numbers of individual names tested at both genus and species level is reported, along with the overall query execution time. A sample web response to a user search on this system is shown in Figure 3.

## Experimental Section

### Materials and Methods

#### Reference database and computing environment

The reference database of notionally correctly spelled names used in the present study was the author's IRMNG database cited above, which at time of testing in May 2013 contained 465,433 genus names and 1,674,319 separate species; a small number of target names flagged misspellings, *nomina nuda* and later usages (duplicate names to be disregarded) were masked during the test operation so as to avoid generation of misleading results, such as would otherwise arise where an input misspelling matches a known misspelled name held in the database, or a stored misspelling masks a true hit during the result shaping stage. Additional details on the content and construction method of this database are available via the IRMNG ‘Frequently Asked Questions’ page [41]. It should be noted that at the present time, IRMNG contains a significant percentage (307,000 of 465,000, or 66%) of genera with no associated species which could therefore be excluded from species-level tests for improved efficiency if desired, however are retained in the production system to cover the possibility that the desired target genus may be present in IRMNG even if the relevant associated species is not currently held. At time of these tests, this database operated on a Dell PowerEdge M610 with two Intel Xeon X56 processors and 128 GB of RAM (processor speed up to 4.4 GHz) running Oracle Enterprise Server 11.2.0 on SuSE Linux Enterprise Server 11 SP2. This specification provides the basis for relevant processing and web response times as reported herein.

#### Training data used for devising heuristics used in the pre- and post- filters

The set of names utilized as sample (training) data for Taxamatch design comprised a set of 641 pairs of species names (input misspelled name plus designated correctly spelled target name) which were identified during routine quality investigations of the CAAB (Codes for Australian Aquatic Biota) marine species database for taxa in Australian waters [6] in 2007; these names, designated the ‘CAAB expert misspellings’ set, are included in File S1 along with the other sets of names used for subsequent algorithm testing. As indicated earlier, lexical patterns in this set of names were used to devise the heuristic rules incorporated into the pre- and post-filters at both genus and species epithet level, and then to manually tune the various filters so that as many false hits as possible were removed without affecting the recall of true hits. A small number of additional adjustments (to two rules only) were subsequently made over the period 2007–2011 when it was discovered that a few additional classes of ‘true’ hits in subsequently supplied data were being discarded prematurely, those errors not being represented in the original sample data, resulting in the minor evolution of Taxamatch from version 1.0 to 1.2 over that period (the newly released version 2.0 is identical to 1.2 but under a different, less restrictive License).

#### Test datasets: species level

1. The ‘CAAB expert misspellings’ set described above (binomials, n = 641; authorities included; errors detected by manual scrutiny after otherwise correctly spelled ‘match’ and ‘no match’ names identified and removed); labelled ‘CAAB’ in relevant tables and charts. Coverage: mostly extant animals (marine species in Australian waters).

2. The ‘Dalcin name pairs’ set included in Appendix III of E. Dalcin’s thesis [12] (binomials, n = 171; authorities omitted; errors as reported by Dalcin); labelled ‘Dalcin’ in relevant tables and data plots. Coverage: mostly extant higher plants (some animals also present, originating from 2 of 5 data sources employed).

3. The ‘CAAB web misspellings’ set as retrieved from CAAB web user logs in 2008 (binomials, n = 2,047; no authorities; errors detected by manual scrutiny after otherwise correctly spelled ‘match’ and ‘no match’ names identified and removed); labelled ‘CAABWEB’ in relevant tables and data plots. Coverage: as per dataset (1) above.

#### Test datasets: genus level

1. The ‘GRIN genera’ set comprising 189 identified misspellings within the set of 28,128 genus names for higher plants downloaded from the GRIN reference taxonomy (USA) [42] in July 2011 (uninomials, authorities included; errors detected by manual scrutiny after otherwise correctly spelled ‘match’ and ‘no match’ names identified and removed); labelled ‘GRIN Genera’ in relevant tables and data plots. Coverage: exclusively extant higher plants.

2. ‘CAAB expert genera’ – being the unique misspelled genus names (only) in the CAAB expert misspellings set as described above (uninomials, n = 116; no authorities); labelled ‘CAAB Genera’ in relevant tables and data plots.

3. ‘CAAB web genera’ – being the unique misspelled genus names (only) in the CAAB web misspellings set as described above (uninomials, n = 853; no authorities); labelled ‘CAABWEB Genera’ in relevant tables and data plots.

4. ‘Dalcin genera’ – being the unique misspelled genus names (only) in the Dalcin misspellings set as described above (uninomials, n = 32; no authorities); labelled ‘Dalcin Genera’ in relevant tables and data plots.

All names in these test datasets, along with their designated ‘correct’ targets, are provided in File S1.

#### Error types

To assist in appraisal of comparative algorithm performance against different classes of error, the following categorization of error types was devised for this study:

- *type 1 error*: single character transformation in a single word, further subdivided into:
  1. *type 1a*: single character insertion/deletion/substitution (except initial character);
  2. *type 1b*: single character transposition (except initial character);
  3. *type 1c*: error at word start (initial character inserted, deleted, substituted, or transposed);
- *type 2 error*: 2-character insertion, deletion, or substitution in a single word;
- *type 3 error*: 2-character transposition, or 3+ character transformations, in a single word;
- *type 4 error* (species only): multi-word error (at least 1 character transformation in both genus and species epithet).

In parallel, errors were classified as either *phonetic* or *non-phonetic* on the basis of application of the ‘Rees near match 2007’ phonetic test, which also allows for common gender mismatches in species epithets.

#### Algorithms used

The algorithms used for the tests reported herein comprise three phonetic algorithms, five dynamic programming algorithms with a range of selected thresholds (totalling 23 variants of the latter on test), plus two versions of Taxamatch (in both normal and ‘no shaping’ modes), as described below. Routines were then created to run these tests largely unattended against all 4,049 names from the seven test datasets over an extended period (several weeks) in May 2013.

1. The previously unpublished *Rees near match 2001* phonetic algorithm, as initially utilized in the CAAB database [6] from 2001 to 2007 and OBIS [7] from 2004 to 2007, applied separately to each word in the case of binomial names. This undertakes the following phonetic transformations: the string is transformed to uppercase; the initial character is retained unchanged (as per Soundex); then selected ‘soundalike’ replacements are performed. The following letters/character groups are equated: AE, OE, E, U and Y are transformed to I; IA and O to A; K to C; MC to MAC; SC, Z to S; H is dropped. Repeated letters (after transformation) are then deleted, i.e. double letters are replaced with single ones. (Note, this is included largely for legacy interest, since it has been superseded in relevant systems by the improved 2007 variant as below).
2. The previously unpublished *Rees near match 2007* phonetic algorithm (including allowance for ‘silent’ leading characters plus additional gender normalization component for species epithets), as developed for later versions of CAAB and OBIS and subsequently incorporated into Taxamatch. This introduces the following additional treatments to those as given above for the 2001 version: selected phonetic transformations are undertaken on the initial character *before* this is quarantined, as follows: leading AE, EA and OE are transformed to E; leading CN, GN, KN and MN to N; leading CT and PT to T; leading CZ to C; leading DJ to J; leading EU to U; leading PH to F; leading PS and TS to S; leading QU to Q; and leading X to Z. Residual transformations are made as per the Rees 2001 version i.e. the initial character is now retained, ‘soundalike’ replacements are carried out including dropping of ‘H’, and repeated letters are deleted. Finally, if the word to be treated is a species epithet (e.g. supplied identified as such, or comprising the second ‘scientific name’ element in a genus+species string), for words now ending in -IS (includes original -is, -us, -ys, -es), -IM (originally -um), -AS (originally -as or -os), the last 2 characters are replaced with -A.
3. *Soundex*, for algorithm description refer e.g. [19]. For the present tests the native Oracle SQL implementation was employed, applied separately to each word in the case of binomial names.
  Note that for testing purposes, in order to obtain maximum algorithm speed, relevant phonetic keys for each of algorithms 1-3 were computed in advance for all target terms and then stored in appropriately indexed database columns along with the original and normalized versions of genus and species names.
4. *Bigrams*. An *n*-gram algorithm was constructed as a custom Oracle PL/SQL function to provide either bigram or trigram testing according to a supplied input value of *n*. This function uses the padded *n*-gram comparison as described earlier and also treats repeated *n*-grams in the same word as separate instances (thus a second instance of the same *n*-gram in the input term will be considered ‘new’ unless matched by a second instance of the same *n*-gram in the target term). The calculated *n*-gram similarity returned by this function is the proportion of common *n*-grams as compared to the arithmetic mean of the total number of *n*-grams in both words. In the tests as reported here, bigrams (*n* = 2) were tested using the similarity thresholds 0.95, 0.90, 0.85, 0.80 and 0.75 as pass/fail criteria.
5. *Trigrams*. This is the case of the *n*-gram test with the value of *n* set to 3. In these tests, trigrams were tested using the similarity thresholds 0.95, 0.90, 0.85, 0.80, 0.75 and 0.70.
6. *Levenshtein Distance* (LD). This was computed using the Oracle PL/SQL implementation of Levenshtein Distance previously published by B. Boehmer [43]. LD tests were undertaken using edit distance (ED) thresholds of 1, 2, 3 and 4.
7. *Damerau-Levenshtein Distance* (DLD). This was computed using the custom PL/SQL MDLD algorithm devised for this study (see next paragraph) with the value for block limit set to 1, which therefore allows only single character transpositions. DLD tests were undertaken using ED thresholds of 1, 2, 3 and 4.
8. *Modified Damerau-Levenshtein Distance* (MDLD). This was computed using the MDLD algorithm as detailed in File S2, with the value for block limit set to 3, which therefore allows double and triple character transpositions in addition to single character transpositions. MDLD tests were undertaken using ED thresholds of 1, 2, 3 and 4.
9. *Taxamatch* (normal mode). This was undertaken using a local implementation of the PL/SQL Taxamatch algorithm described herein, with result shaping engaged as recommended for normal use.
10. *Taxamatch (no shaping)*. This was undertaken with result shaping omitted, as might be used in situations where a maximum degree of recall is required, at the expense of (potentially) additional false hits.

The tests on execution time using a small subset of input names were carried out as a search against the entire target dataset, but as will be apparent from those data, in the case of the dynamic algorithms, long execution times of between 180 and 1,011 seconds per input name were encountered at species level. Therefore, for the bulk precision and recall testing required for this study, for these algorithms some modest pre-filtering was undertaking in order to reduce overall processing time: first, in the genus case, target genera greater than 4 characters longer or shorter than the input genus were not tested (and for species, target species of such genera), and second at species level, target species scoring less than 0.5 on the bigram similarity test were omitted from testing by the other dynamic algorithms on the basis that they would not be sufficiently similar to be worth further consideration. By these means, the overall test regime for all input names was reduced from potentially several months to around 3 weeks.

#### Subsetting of input names for detailed performance testing

For the individual run time data reported per algorithm, a ‘randomized’ subset of names was obtained by selecting the 500^th^, 1,000^th^, 1,500^th^, 2,000^th^ and 2,500^th^ species name of 2,859 names across all input data sets (sorted alphabetically) and then rearranging these for presentation by name length, and for genera, the 200^th^, 400^th^, 600^th^, 800^th^ and 1,000^th^ name of 1,190 names, similarly re-sorted.

#### Metrics and methods used for reporting algorithm performance

The performance metrics of *recall* and *precision* have been described above. For algorithm *effectiveness* the *F*_1_ measure is reported, the harmonic mean of precision and recall, both on a 0–1 scale, calculated as per [29], which facilitates potential comparison with other studies since this is a commonly used measure of general algorithm performance in the wider information retrieval domain. In the present context, the relevant effectiveness values enable more straightforward inter-algorithm or inter-dataset comparisons to be made and in addition, when determined at a range of thresholds, can be used to define a notional ‘best’ setting, i.e. where the maximum *F*_1_ value is achieved.

Algorithm *efficiency* is represented here via the proxy of execution time on standard computer hardware. For Taxamatch (both normal and no shaping variants), execution times were determined using a previously constructed PL/SQL routine as accessed via the IRMNG web query interface, which as shown in Figure 3 includes a report of execution time as well as the relevant near match results.

## Results and Preliminary Discussion

### Comparative algorithm performance

**Figure 4:**
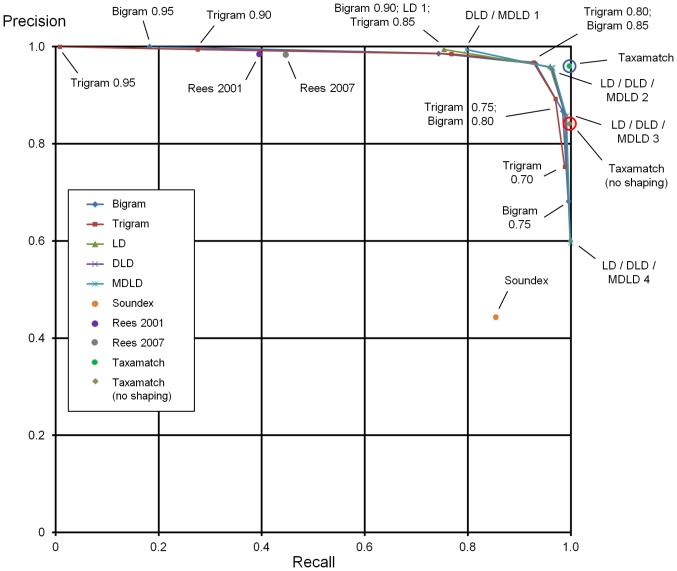
Species level precision:recall curves for all algorithms tested, as binomial names, means of three datasets (data from **Table 1**). Performance of five dynamic algorithm variants, three phonetic algorithms plus two Taxamatch variants using the three available misspellings datasets for species, at a range of thresholds in the case of the dynamic algorithms. Performance of Taxamatch and Taxamatch ‘no shaping’ variant are highlighted in blue and red circles, respectively. Data values closest to 1,1 (upper right corner) indicate best performing setting (maximum effectiveness) for a given algorithm. (Note at this scale, curves for certain variants i.e. LD, DLD lie behind others i.e. MDLD in some places, similarly for bigrams versus trigrams).

**Figure 5:**
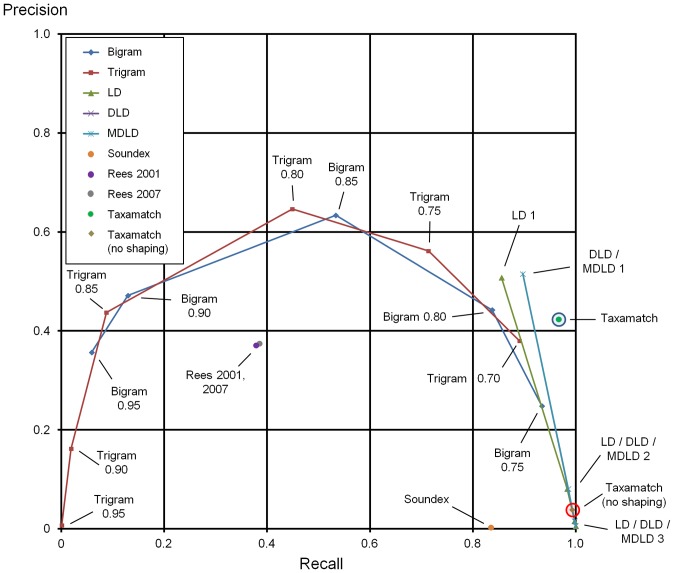
Genus level precision:recall curves for all algorithms tested, means of four datasets (data from **Table 1**). Performance of five dynamic algorithm variants, three phonetic algorithms plus two Taxamatch variants using the four available misspellings datasets for genus names only, at a range of thresholds in the case of the dynamic algorithms. Performance of Taxamatch and Taxamatch ‘no shaping’ variant are highlighted in blue and red circles, respectively. As in previous Figures, data values closest to 1,1 (upper right corner) indicate best performing setting (maximum effectiveness) for a given algorithm.

**Figure 6:**
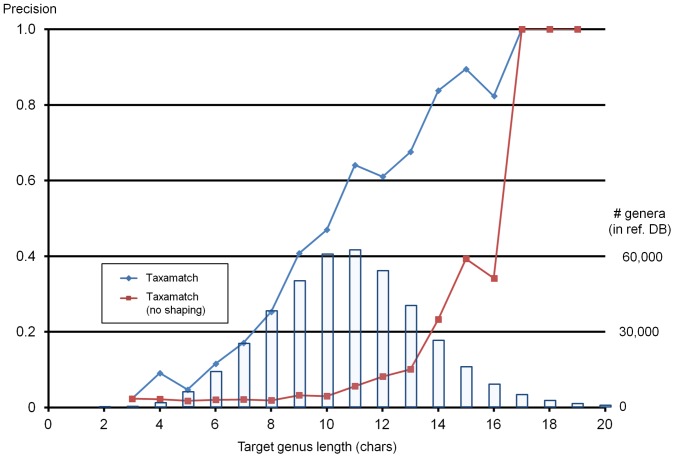
Genus-level precision values for Taxamatch (both variants) as a function of genus length. Data shown are from the four genus-only datasets combined. Superimposed columns indicate distribution of target genus names in the reference database as a function of genus length. The two most frequent lengths of genera in the reference (IRMNG) database at this time (n = 62,228 and 64,062 for genus lengths 10 and 11 characters, respectively) together comprise 29.7% of all genus names in the database, excluding known misspellings, *nomina nuda*, later usages and virus genera.

File S1 gives numbers of true and false hits for all 2,859 input species names and 1,190 genus names, from 7 datasets as described above, tested against the IRMNG reference database as at May 2013 using all 28 algorithm variants. Summary data for algorithm performance, as average recall, precision and effectiveness, against both species names and genera are presented in Table 1, with precision:recall plots based on these data in Figures 4–5. Figure 6 documents Taxamatch performance against uninomials (genus names) as a function of word length.

**Table 1:** Overall algorithm performance as recall, precision and effectiveness (*F*_1_ measure) – mean values of all datasets tested (3 for species, 4 for genera).

		SPECIES			GENERA		
Algorithm type	Algorithm (+ threshold where relevant)	Mean recall	Mean precision	Mean *F*_1_	Mean recall	Mean precision	Mean *F*_1_
Phonetic	Rees 2001	0.395	0.985	0.560	**0.385**	**0.375**	0.379 *
	Rees 2007	**0.447**	**0.983**	0.613 *	0.379	0.371	0.374
	Soundex	0.854	0.443	0.584	0.835	0.003	0.006
Dynamic (*n*-gram)	Bigram 0.95	0.182	1.000	0.295	0.059	0.357	0.100
	Bigram 0.90	0.743	0.986	0.841	0.129	0.472	0.202
	Bigram 0.85	**0.932**	**0.965**	0.947 *	0.533	0.633	0.576
	Bigram 0.80	0.984	0.868	0.922	**0.838**	**0.442**	0.578 *
	Bigram 0.75	0.995	0.682	0.809	0.934	0.248	0.392
	Trigram 0.95	0.008	1.000	0.015	0.001	0.007	0.001
	Trigram 0.90	0.275	0.994	0.415	0.019	0.162	0.034
	Trigram 0.85	0.768	0.985	0.858	0.088	0.437	0.145
	Trigram 0.80	**0.927**	**0.967**	0.946 *	0.449	0.646	0.527
	Trigram 0.75	0.970	0.893	0.929	**0.714**	**0.562**	0.627 *
	Trigram 0.70	0.988	0.752	0.854	0.891	0.380	0.532
Dynamic (edit distance)	LD 1	0.754	0.994	0.855	**0.856**	**0.508**	0.635 *
	LD 2	**0.959**	**0.959**	0.959 *	0.983	0.081	0.149
	LD 3	0.988	0.862	0.921	0.998	0.016	0.032
	LD 4	0.999	0.603	0.751	1.000	0.006	0.012
	DLD 1	0.797	0.994	0.882	**0.897**	**0.515**	0.652 *
	DLD 2	**0.963**	**0.956**	0.959 *	0.985	0.081	0.148
	DLD 3	0.989	0.857	0.918	0.998	0.016	0.032
	DLD 4	0.999	0.596	0.746	1.000	0.006	0.012
	MDLD 1	0.797	0.994	0.882	**0.897**	**0.515**	0.652 *
	MDLD 2	**0.965**	**0.956**	0.960 *	0.986	0.081	0.148
	MDLD 3	0.991	0.857	0.919	0.998	0.016	0.032
	MDLD 4	0.999	0.596	0.746	1.000	0.006	0.012
Custom	Taxamatch	**0.997**	**0.960**	0.978 *	**0.967**	**0.423**	0.587 *
	Taxamatch (no shaping)	0.997	0.841	0.912	0.993	0.039	0.074

Results are averaged from values for all datasets tested, i.e. datasets are accorded equal weight independent of dataset size. Asterisks (plus entries in bold type) indicate best performing (most effective) setting of individual algorithm variants (separately for species and for genera alone), based on highest *F*_1_ value.

From Table 1 and Figure 4 it is clear that at species level, each of the dynamic algorithms perform quite well (although outperformed by Taxamatch) at their ‘best’ i.e. most effective settings (highest *F*_1_) in that they quite closely approach the ‘ideal’ value (precision = 1, recall = 1) before dropping off as thresholds are widened; however it is noteworthy that is it not possible to retrieve the last 3–5% of true hits without a corresponding decrease in precision. By contrast, the Rees 2001 and 2007 phonetic algorithms show high precision but only moderate recall, while Soundex has better recall but poorer precision. Of the dynamic algorithms, bigrams and trigrams perform well (good precision) up to around 93% recall and the edit distance based algorithms LD, DLD and MDLD up to around 96% recall before precision decreases markedly. Taxamatch maintains high precision even at 99.7% recall while the ‘no shaping’ variant provides the same displayed overall recall in this tests (in fact 1 additional hit is returned at species level) with around a 12% drop in precision overall.

At genus level (Figure 5) the situation is somewhat different, principally because the text strings involved are shorter (which has an influence on the *n*-gram similarity measures in particular) and also many genus names, including potentially non-target ones are quite similarly spelled, particularly shorter names (see below). The *n*-gram based tests for genera alone show low recall at narrow thresholds and precision is also affected since this cannot be high unless at least some true hits are returned. Recall is substantially better for the edit distance tests at narrow thresholds but precision decreases rapidly when the edit distance threshold exceeds ED 1. The normal version of Taxamatch does provide improved performance but in this case fails to retrieve up to 4% of true hits at genus level; these hits can largely be retrieved by the ‘no shaping’ variant but with the loss of a significant amount of precision. (In operation at species level, result shaping is not applied until epithets have been tested which means that no near match genera will be rejected if they possess an accepted near match species).

A significant contribution to the comparatively poorer precision results for genera alone as compared with species names (as binomials) is the fact that many short genus names can be lexically quite similar to other correctly spelled genera. As an example of the latter, in IRMNG at the present time, within ED (as MDLD) 1 of the (correctly spelled) genus name *Homo* (humans and their nearest fossil relatives) are two other unique names/genera namely *Hoho*, an amphipod and *Homa*, a hemipteran, while within ED 2 are an additional 68 genera (61 unique names) (using the pre- and post-filtering rules employed in Taxamatch this set of ED 2 matches is reduced to only 3, namely two instances of *Hama*, representing a moth and a fossil fish, plus *Homia*, a beetle). Extending the permitted edit distance to 3 (which would not be possible within Taxamatch on account of the ‘minimum 50% good characters’ rule) would return a further 960 genera (744 unique names) for this 4 character word. By contrast, with a randomly selected longer genus name – for example *Catharacta*, an example correctly spelled target name from this study, length 10 characters – equivalent values are much lower, namely 0 unique names/genera at ED 1, 5 unique names/genera at ED 2 and just 26 genera (22 unique names) at ED 3, prior to any Taxamatch-associated filtering. This suggests that the poorest precision (largest numbers of false hits) will be encountered with short input or target names, a characteristic which is borne out in practice as shown in Figure 6 using Taxamatch precision data as examples.

From these data it can be seen that with result shaping employed i.e. the ‘normal’ mode, genus precision is poor (e.g.<0.4) until target genus length reaches around 9 characters, and thereafter steadily improves. Fortunately, as can be seen from the name frequency data also included in Figure 6, the most commonly occurring genus names in the reference database (approximating the whole of biology) are in the 9–12 character range where precision is at least acceptable (varying from around 0.4 to 0.6) while for longer names, precision is better again (by contrast, the no shaping variant performs relatively poorly in this respect except in the case of very long names).

Returning to the inter-algorithm comparison data for genera, Soundex is poor in the precision metric for a different reason, namely that the same Soundex code (key) can be shared by multiple genera, in many cases independent of term length since typically the Soundex key is drawn from the leading 4–8 characters only. As an example, at the present time the Soundex key ‘P232’ is shared by 2,953 genera in the IRMNG reference database ranging in length from 6 to 24 characters, commencing in the shortest case with *Pectis*, *Pictus* and *Pistus* and ending in the longest with *Pseudoglossodiplostomum*, *Pseudoschizorhynchoides* and *Pseudocoeliodidymocystis*, indicating that while in popular descriptions this algorithm is classified as ‘phonetic’, in practice the resulting near matches in the taxonomic domain certainly do not qualify as a set of phonetic equivalents. In contrast to Soundex, the Rees 2001 and 2007 algorithms are considerably more selective for phonetic matching: for example the maximum number of distinct genera sharing the same Rees 2007 phonetic key (in this instance ‘RISA’) is 36, with the shortest *Risa* and *Rusa*, and the longest *Reuschia*, *Ruehssia* and *Ruyschia*. At genus level, the recall of the Rees 2007 near match algorithm is slightly worse than that for the Rees 2001 algorithm, a result which was not anticipated on account of the additional phonetic transforms of the initial character/s incorporated into the 2007 version which are intended to improve rather than degrade matching performance. Inspection of the names in question reveals that this discrepancy is accounted for by the substitution of ‘F’ for a leading ‘Ph’ in relevant cases, which now means that the latter will not match ‘P’ (with no ‘h’ following) and vice versa. However this step is retained since in some cases it would notionally be useful, for example equating ‘Facus’ as a phonetic match for *Phacus* and so on.

### Algorithm efficiency

Comparative algorithm efficiency, as represented by indicative overall query execution times for five selected input species and five genus names, is shown in Tables 2 and 3 (which as noted in the Materials and Methods section, contain data without any additional pre-filtering of target names employed elsewhere to obtain shorter processing times). Also reported are the number of names passing both the genus or species pre-filter stages (as applicable) in Taxamatch operation for each query, giving indications of the selectivity values associated with each, which can then be compared with the hypothetical values discussed earlier in the section on algorithm design.

**Table 2:** Sample execution times by algorithm: selected species tests, tested against all IRMNG species (1.67 m names) in May 2013.

**Input name**	*Peronella lesueri*	*Fusinius undulatus*	*Sigonus caraliculatus*	*Lutjanus carponotatuus*	*Cephaloscyllium fasciatumm*	
**Input name length (chars)**	17	18	21	22	26	
**Target name**	*Peronella lesueuri*	*Fusinus undulatus*	*Siganus canaliculatus*	*Lutjanus carponotatus*	*Cephaloscyllium fasciatum*	
**Algorithm**	**Execution time (seconds)**					Mean execution time per name-name comparison
Soundex	0.027	0.030	0.014	0.030	0.005	<0.0001 ms
Rees 2001	0.016	0.035	0.024	0.009	0.051	<0.0001 ms
Rees 2007	0.027	0.020	0.008	0.027	0.051	<0.0001 ms
Bigram 0.85	180.3	187.7	206.6	212.7	246.1	0.129 ms
Trigram 0.80	215.3	223.2	243.7	251.1	285.4	0.152 ms
LD 2	221.2	231.4	265.3	280.0	329.9	0.166 ms
DLD 2	421.2	427.7	497.1	522.3	640.5	0.314 ms
MDLD 2	645.6	662.5	773.6	814.1	1,011.4	0.488 ms
Taxamatch	1.487	1.067	0.982	1.530	1.677	0.257 ms *
	**Taxamatch pre-filter operation:**					**Mean efficiency (as reduction in total tests required)**
Taxamatch genera passing pre-filter (of 465,433)	15,751	4,640	2,802	4,306	5,341	98.6%
Taxamatch species passing pre-filter (of 1.67 million)	900	329	289	658	35	99.6%

Taxamatch mean execution times per name-name comparison (indicated with asterisk) are calculated against the number of names actually tested (many names being excluded by operation of relevant pre-filters).

**Table 3:** Sample execution times by algorithm: selected genus tests, tested against all IRMNG species (465 k names) in May 2013.

**Input name**	*Schuetta*	*Euathronia*			*Leucolaena*	*Cathharacta*		*Pastinachhus*	
**Input name length (chars)**	8	10			10	11		12	
**Target name**	*Schuettea*	*Euarthronia*			*Leucolena*	*Catharacta*		*Pastinachus*	
**Algorithm**	**Execution time (seconds)**								Mean execution time per name-name comparison
Soundex	0.003		0.005	0.004			0.012	0.037	<0.0001 ms
Rees 2001	0.001		0.002	0.002			0.002	0.002	<0.0001 ms
Rees 2007	0.001		0.001	0.001			0.002	0.002	<0.0001 ms
Bigram 0.80	23.4		27.4	27.3			28.2	29.2	0.058 ms
Trigram 0.75	28.6		31.8	31.3			33.0	35.0	0.069 ms
LD 1	18.6		21.2	21.2			23.0	25.5	0.047 ms
DLD 1	32.4		38.2	39.3			43.6	44.8	0.085 ms
MDLD 1	42.3		54.5	56.4			60.8	62.4	0.119 ms
Taxamatch	1.164		1.557	1.105			0.836	1.575	0.565 ms *
	**Taxamatch pre-filter operation:**								**Mean efficiency**
Taxamatch genera passing pre-filter (of 465,433)	923		9,136	2,480			1,091	8,785	99.0%

Taxamatch mean execution times per name-name comparison (indicated with asterisk) are calculated against the number of names actually tested (many names being excluded by operation of relevant pre-filter).

These results show that, as expected, the three phonetic algorithms are extremely fast in the present context (below 0.0001 ms per name-name comparison, less than 0.1 seconds to test a single input name against all target names, whether as species binomials or genera). The *n*-gram and edit distance based algorithms are considerably slower in the implementations on test, for a binomial term (species name) varying from 180 to over 1,000 seconds and for single term (genus name) varying from 23 to 62 seconds, principally according to input name length and algorithm type (note that in the genus tests, input names are tested against a smaller reference set, i.e. 28% of the number of targets, as compared with the full species dataset, and are faster for that reason, in addition to the shorter average target term length). Individual execution times for these tests increase with algorithm complexity i.e. bigrams to trigrams, LD through DLD to MLD and also roughly linearly with input term length against these fixed sets of target names.

The Taxamatch tests achieve their intended goal of much faster execution time overall than the unmodified dynamic algorithms, in line with the anticipated efficiencies introduced earlier in the section on algorithm design. In that section it was suggested that a genus pre-filter efficiency of 90% and an overall species-level efficiency of 99.95% might result in ‘acceptable’ performance against even large reference datasets; mean values from this (limited) set of tests indicate a range between 96.6% and 99.8% for the genus pre-filter, and an high overall species-level efficiency (99.6%), permitting completion of the algorithm workflow in between 0.8 and 1.7 seconds, varying chiefly according to characteristics of the input genus name in particular. Using the example names as tested in Table 2, numbers of genus names requiring the dynamic test varied from under 3,000 to under 16,000 (a saving of between 449,000 to 463,000 genus tests alone) and the residual number of species epithets tested varied from 35 to 900, a trivial number in relation to the almost 1.7 million names in the reference database, an indication of the efficiencies gained from operation of the Taxamatch pre-filters at the respective levels.

One other aspect worthy of comment in the present set of tests is that the reference database used is arguably fairly complete at genus level (missing perhaps a maximum of 10% of names for all biota, refer [41]) despite being considerably less complete – probably missing 50% or more of all published names – at species level. Because the genus pre- and post-filters have the most influence on overall algorithm performance, it is therefore likely that the performance illustrated above may not degrade substantially against even more comprehensive reference datasets since the addition of more species without the requirement to add further associated genera would have a relatively modest impact on overall execution time.

### Algorithm performance by error type

**Figure 7:**
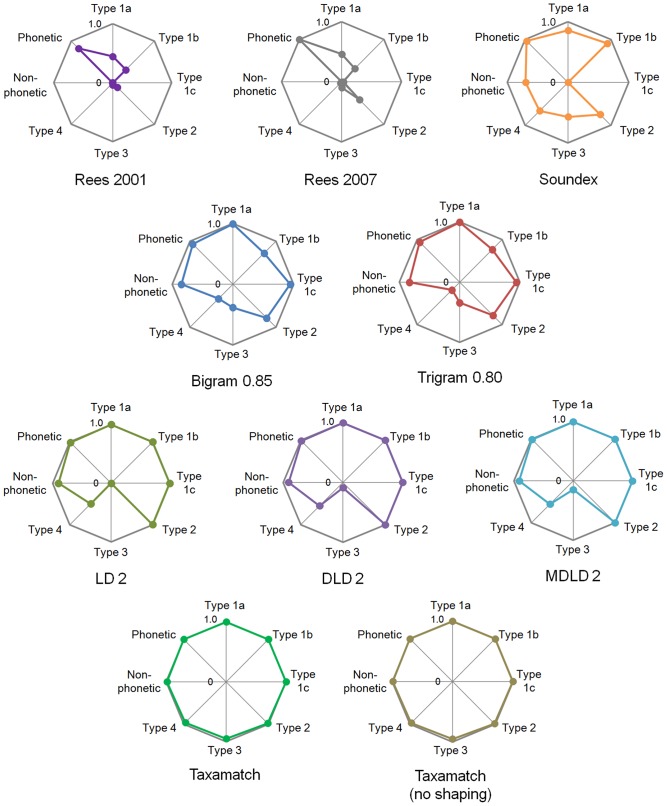
Species-level recall by error type for each algorithm tested in the present study. Values for the dynamic/variable threshold algorithms are derived from their ‘best’, i.e. most effective settings, for all species data pooled (data from Table 4).

An issue with reporting aggregate performance data across whole datasets (or all datasets combined) is that the results will tend to be dominated by performance against the commoner error types. However, from an operational perspective it is equally desirable that less common error types (such as a mistake in the leading character of a word, lexically more complex errors, or simultaneous errors in both genus and species epithet) still result in the intended target being returned where available (i.e., no false negatives in these cases). To investigate this aspect of algorithm performance in more detail, the pooled species data were disaggregated by error types 1a through 4 as defined in the Materials and Methods section, and also separately classified into either phonetic or non-phonetic errors; in this case performance is reported as recall alone since as stated above, the avoidance of false negatives is our main interest. (Data for genera are not presented but in general mirror those for binomials, except that type 4 errors are not applicable in that case); results for this analysis by algorithm and error type are presented in Table 4 and Figure 7.

**Table 4:** Algorithm recall (on 0–1 scale) by error type for all species data pooled (n = 2,859); dynamic algorithms used at their ‘best’ settings (maximum *F*_1_ value) as determined from Table 1.

Algorithm	Recall by error type							
	Type 1a (n = 1,926)	Type 1b (n = 125)	Type 1c (n = 36)	Type 2 (n = 504)	Type 3 (n = 102)	Type 4 (n = 166)	Phonetic - all (n = 1,171)	Non-phonetic - all (n = 1,688)
Rees 2001	0.446	0.312	0.000	0.111	0.039	0.000	0.824	0.006
Rees 2007	0.460	0.312	0.028	0.419	0.098	0.028	1.000	0.000
Soundex	0.858	0.912	0.000	0.748	0.569	0.663	0.965	0.700
Bigram 0.85	0.996	0.728	0.944	0.786	0.382	0.337	0.938	0.851
Trigram 0.80	0.996	0.768	0.944	0.782	0.343	0.181	0.938	0.835
LD 2	1.000	1.000	1.000	1.000	0.000	0.494	0.985	0.900
DLD 2	1.000	1.000	1.000	1.000	0.088	0.554	0.987	0.910
MDLD 2	1.000	1.000	1.000	1.000	0.147	0.554	0.987	0.914
Taxamatch	1.000	1.000	1.000	0.984	0.951	0.964	1.000	0.989
Taxamatch (no shaping)	1.000	1.000	1.000	0.984	0.951	0.970	1.000	0.989

These results demonstrate that of all algorithms on test, only the two Taxamatch variants achieve consistently good recall across all error types in the available test species datasets. The Rees 2007 near match algorithm is improved over the original 2001 version in particular against type 2 errors (which include a component of potential gender mismatches in species epithets) and phonetic errors in general (in equivalent genus-only data the differences are less marked since the gender mismatch component is not required). Type 1c errors (mismatch at word start) in general defeat all of the phonetic algorithms, since (with the exception of special cases built into the Rees 2007 algorithm, not extensively represented in the present test data) these are constrained to require a match on the leading character. The *n*-gram variants perform poorly against more severe errors (types 3 and 4) at these ‘best’ settings and sub-optimally against type 1b and type 2 errors (transpositions and 2-character errors), while the edit distance based tests (again at the moderate thresholds at which they are most effective) perform somewhat better against the simpler errors (types 1 and 2) but less well against more severe errors of types 3 and 4; a small improvement against type 3 errors is seen in the progression from LD through DLD to MDLD. By contrast, both Taxamatch variants show consistent high recall levels against all error types and are clearly superior to the other algorithms on test in this regard, particularly in respect of the most ‘difficult’ i.e. type 3 and type 4 errors which include multi-character errors, 2 character transpositions, and simultaneous errors in both genus and species epithet.

### Inter-dataset comparisons

**Figure 8:**
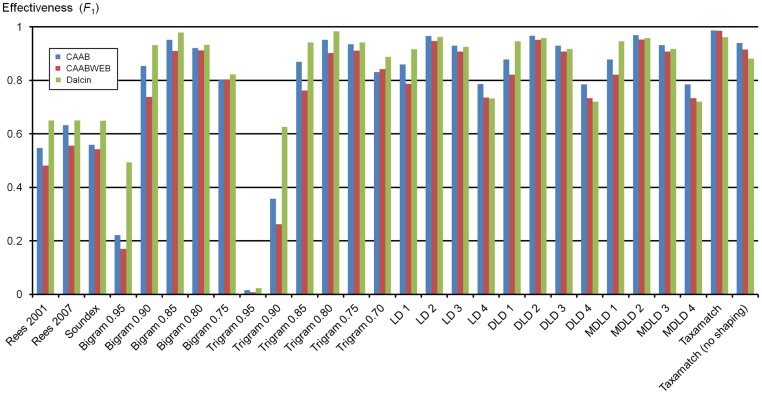
Species-level effectiveness (*F*_1_) for all algorithms at all settings, disaggregated by dataset. Note variation in *F* **_1_** value with varying threshold setting for each of the dynamic algorithms, with peak at setting 0.85 (bigrams), 0.80 (trigrams) and ED 2 for the LD, DLD and MDLD tests.

**Figure 9:**
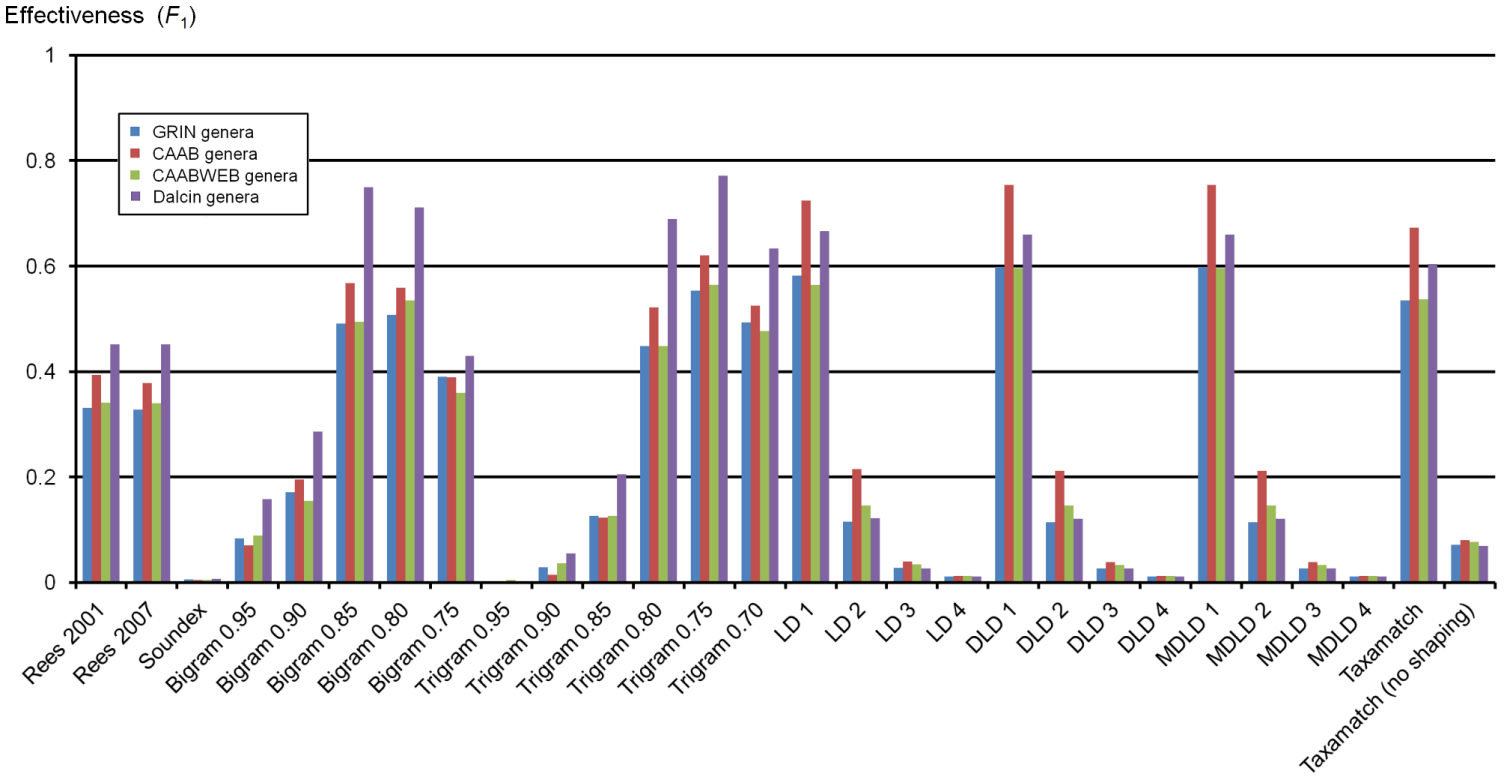
Genus-level effectiveness (*F*_1_) for all algorithms at all settings, disaggregated by dataset. Note variation in *F* **_1_** value with varying threshold setting for each of the dynamic algorithms, with peak at setting 0.80 (bigrams), 0.75 (trigrams) and ED 1 for the LD, DLD and MDLD tests. Taxamatch values are slightly depressed by this metric compared with some of the ‘best’ dynamic algorithms on account of sacrificing some precision for close to 100% recall (cf. Figure 5).

Since the main algorithm design stages were undertaken using a single set of training data (the CAAB expert misspellings set) it is appropriate to investigate any biases which may inadvertently have been included as well as the inherent variation in available sets of misspelled names, by examining algorithm performance by dataset. These results are presented with the Y-axis representing effectiveness (*F*_1_) per dataset using each setting of every algorithm employed for species names and for genera alone in Figure 8 and Figure 9, respectively.

Inspection of these data suggests that at species level, the CAAB expert misspellings set used as sample data is comparable with the other datasets (at genus level the algorithms perform rather better against this than the other sets, suggesting possibly the presence of more severe errors in the latter). Selection of the best (most effective) setting for each algorithm is broadly unaffected by choice of dataset at both species and genus level. The CAAB web misspellings dataset proved a somewhat more severe test for all algorithms than the others, returning the lowest overall effectiveness for most algorithms although Taxamatch performance was equal best for this set (at species level). In general there appear to be no particular biases introduced by the particular choice of sample data which give some confidence in the more general applicability of the various rules and thresholds derived from use of this source for the initial algorithm development.

## General Discussion and Conclusions

### Taxamatch performance against design goals

Overall it can be seen that by adopting the strategies outlined in the theoretical portion of this paper, Taxamatch achieves its design goals of high recall, high precision and acceptable efficiency (in this instance, execution time of generally less than 2 seconds per input name against a large reference database) and does this to a more effective degree than the other algorithms on test, against a full spectrum of error types, especially the more ‘difficult’ type 3 and 4 errors. Phonetic algorithms are very fast in execution time but fail to retrieve non-phonetic errors except (to a degree) in the case of Soundex, however the latter performs poorly in the area of precision (in other words this algorithm may return large sets of candidate near matches, however many of these can be lexically quite distant from the desired target term). The dynamic algorithms used at their maximum effectiveness settings perform well against simple errors but are defeated by more complex error types and in addition, are almost unusably slow against substantial sets of target data such as thousands or millions of names without additional pre-filtering to avoid testing all names, along the lines introduced with Taxamatch.

### Options to further reduce false hits with Taxamatch

In the present study Taxamatch, even in the no shaping mode, returned few false positives at species level but an increased number for genera alone, especially for short words, despite application of the pre- and post-filters at genus level. Figure 6 indicates that precision can be 0.1 (9 false hits out of every 10) or less for 4 and 5 character words, rising only to 0.2 for 7 character words and not passing 0.5 until 10 character words are reached. If these values are considered sub-optimal then two approaches are conceivable: one is to further tighten the operation of the filters at genus level (a possible area for future investigation) and the other is to use a smaller reference database which omits may non-target terms, for example comparing only insects with insects, higher plants with higher plants, and so on. Such options are currently offered in the Taxamatch instance deployed in the current IRMNG web interface [40] where they can prove very useful in further reducing false hits. However, they will not be 100% infallible in a few cases where higher taxonomy is uncertain, as is the case with some taxa e.g. fossil forms possibly representing either Porifera or Bryozoa, or in groups where relevant higher classification is unstable (such as the uncertain area between single celled algae and Protista or Chromista, and similarly with certain current or ex-current fungal groups).

### Analysis of residual true hits not returned using Taxamatch (false negatives)

The mean values for Taxamatch recall presented in Table 1 for species do not quite attain the ‘ideal’ value (i.e. 0.997 instead of 1.000) on account of a small number of designated target names not retrieved from a single data source, the CAAB web misspellings set, for which 19 true hits were not returned out of a total 2,047 (18 for the no shaping variant). Further investigation showed that 6 of those names exceeded either the combined or species epithet alone maximum edit distance threshold (ED 4) and can arguably be discounted as severely misspelled. One input name (*Aleura scripta*) failed to match its designated target, *Aleuron scriptor* (a moth in the family Sphingidae) on account of being masked by the lexically closer (ED 2 versus ED 4) designated false hit *Asura scripta* (also a moth but in a different family) in the normal Taxamatch mode; in the ‘no shaping’ mode the correct match was returned along with two more false hits, *Alesia striata* and *Asura striata*. The residual 12 true hits not returned were rejected in either the genus pre-filter (1 case), the genus post-filter (2 cases) or species post-filter (9 cases) on account of possessing lexical characteristics not present in the sample data used for Taxamatch filter design. A question then arises as to whether to relax the rules in question so as to accommodate these rare cases, which would almost certainly reduce algorithm precision for all searches, or whether to leave the rules on their present settings and accept that a small percentage of unusual misspelled names may be rejected. At present the recommendation is for the latter of these two options but this can be reviewed through time as desired.

### Further improving Taxamatch execution time

As shown in Table 4 and on the basis of the author's experience over a 5+ year period, Taxamatch operation in the current system is probably acceptable for single name queries (between 1 and 2 seconds per input species name, slightly less for genera alone) but further improvement would be desirable, particularly in the case of large scale data deduplication: for example testing 2 million species names against each other currently requires several weeks of continuous operation (2 m×2 m names at average 1.5 seconds per test would take 833 hours or 34.7 days to complete). To this end, some potential avenues for future speed enhancement can be envisaged and might include:

- increasing the speed of the MDLD comparison (for example by porting to a faster programming language, deploying on a more powerful platform, and/or other enhancements);
- splitting the phonetic and edit distance portions of the algorithm, i.e. first seeking a phonetic match, and if found omitting the remaining tests (together with any additional near matches they might return);
- restricting name-name comparison to the names in the same taxonomic group, as previously discussed in the context of reducing false positives, however here to reduce execution time;
- drastically reducing the set/s of names to be tested – for example restricting genus testing to names where either genus or species epithet is a phonetic match;
- further streamlining/optimizing the internal algorithm implementation if possible, via external code review and/or shared development.

Of these, the first and last comprise hardware and software optimization and incremental improvement, and may be expected to happen through time as a normal process of ongoing development, though it is unlikely that more than perhaps 50% improvement in overall execution time would be anticipated unless the software were deployed on substantially faster hardware. The second option would be quite feasible if desired and would lead to some searches operating very rapidly (perhaps 50%) while the remainder would be unaffected; it would however have the potential side effect of masking non-phonetic matches in the presence of phonetic ones, which could be undesirable in some use cases. The third option should be acceptable in most cases, except where a name has been mis-classified, and could save from approx. 60% to over 90% of required tests, according to the group in question, while the fourth option has been found to be highly effective in bulk species-level tests, covering over 99% of cases in practice, eliminating almost no true hits, however will not provide an acceptable alternative for genus-only tests since non-phonetic errors in genera alone will not be detected. This facility has been available on the present Taxamatch-enabled IRMNG web search interface since 2008, where it is designated ‘Taxamatch rapid’. In use, taking for example the misspelled input name *Sigonus caraliculatus* (example from Table 2, selected because it represents a mid-range name length in characters) the following comparative results are obtained:

- normal Taxamatch operation (data from Table 2): ED test is applied to 2,802 genera+289 species, execution time = 0.982 seconds;
- restrict testing to names from the same taxonomic group, in this example: ‘superclass’ Pisces (option c above): ED test is applied to 193 genera +64 species, execution time = 0.536 seconds;
- restrict genus testing to names where either genus or species epithet is a phonetic match (option d above, ‘Taxamatch rapid’ mode): ED test is applied to 1 genus+44 species, execution time = 0.022 seconds.

These data certainly suggest that improved run times would be available using the above principles where required, and for example might be of value in the cases of either bulk matching of user-supplied lists of input names without an extensive wait period, or for extensive internal data deduplication runs at species level in particular, where the failure to retrieve a very small number of true hits would be an acceptable trade-off.

Two other approaches have been proposed for improving the operational performance of the dynamic comparison methods discussed here. For *n*-gram comparisons, *n*-grams for all target terms can be generated in advance, stored (at the cost of minor additional database space) and indexed for fast access [44], which then reduces the overall computational cost of each individual *n*-gram comparison if this is required (in the present context this is not an issue since edit distance is the preferred approach for the bulk of term comparisons). For Levenshtein distance or variants thereof, the use of a *Levenshtein automaton* has been described in [45]; in essence, running the automaton (based upon the input term as starting point) generates the set of all possible new terms which can be created by any potential insertion, deletion, substitution or transposition operation to the required maximum edit distance, and this set is then intersected with all available target terms as an exact match process. If the alphabet to be used is kept small (e.g. the 26 a–z characters only, discounting case) one can see that, for example, with a 4-character word there are 130 (5×26) potential insertions, 4 potential deletions, 100 (4×25) potential substitutions and 3 potential single character transpositions, a total of 237 new words that could be formed at ED 1, and for a 10-character word the equivalent number is 555, still a relatively trivial set to test by exact matching. The penalty with this approach is that it scales roughly factorially, in that if there are in the order of 500 ED 1 transformations for a 10-character input term there will be in the order of 250,000 (500×500) ED 2 transformations, 125,000,000 (500×500×500) ED 3 transformations and so on, and first computing these and then testing either all or even a subset of them, for example as suggested in [46], may not produce a significant time saving over a standard run time Levenshtein approach at edit distances greater than 2, or more particularly with Taxamatch which further limits the degree to which the dynamic tests are required.

### Extending Taxamatch operation to ranks not covered above

The basic Taxamatch algorithm presented here shows how the algorithm addresses the issue of matching generic names, species names as binomials and the authority portions of taxonomic names. The algorithm can be extended as required to cope with uninomials at other ranks (i.e. higher than genus level), subgenus names (for example supplied in parentheses following a genus name) and infraspecific taxon names, for example by searching zoological subgenera against a the same database of generic names (the two are interchangeable in zoology) or in botany, against a separate, smaller list of subgenera. (Infraspecific taxon names would most likely be searched against lists of known infraspecific names only, which would be a logical recursive step to follow the search at species epithet level). Hybrid formulae for species, where the genus and species names of both parents may be included, can similarly be accommodated with little difficulty provided that appropriate parsing can be done in a controlled and effective manner.

### Appraisal of alternative (non-Taxamatch) ‘near match’ approaches currently offered in the taxonomic domain

Having investigated in some detail the performance of different algorithmic approaches in comparison with Taxamatch, it is now possible to offer comments on the small range of near match options currently offered by other systems. Soundex, as offered by systems [3], [4] and [5] mentioned in the Introduction, is likely on the basis of present tests to miss around 15% of desired correct targets at both genus and species levels, in particular excluding those where the leading character does not match, and at the same time produces quite large numbers of false hits; in addition, Soundex will not discard likely non-matches on the basis of word length and will frequently suggest some fairly bizarre matches as noted above. Taxonome [11] uses a modified *n*-gram test (trigrams, with two padding characters at the beginning only) and a default internal threshold of 70% similarity, which can be altered as desired in the source code, further constrained to match on the leading 3 characters of genus so as to avoid testing all names. The nearest equivalent in these tests (Trigrams at 0.70 thresholds, padded both ends) did return 98.8% of species targets on average (89% for genera), however without additional pre- or post-filtering will tend to produce elevated levels of false hits. As presently deployed in Taxonome, ‘correct’ targets with a difference in the leading 3 characters of the genus name will also be eliminated from the near matches offered; in the present test data, this would apply to 252 of 2,860 misspelled species and to 215 of the 1,191 genera, values of 8.8% and 18.1%, respectively.

The standard Levenshtein Distance, as employed in the GRIN Taxonomic Nomenclature Checker [8] with a user-selectable threshold of edit distance, will perform adequately against all but the transposed syllable error class, although it will catch these at threshold ED 4, provided that the user starts with a reasonably tight initial threshold setting e.g. ED 2 and then manually widens this and re-runs the search in the event that no hits are returned. However as with *n*-grams, LD will be susceptible to elevated levels of false hits in the absence of a mechanism to address these. PlantMiner [9] and Taxonstand [10]) both use ‘agrep’ which is very fast according to its creators (algorithm not tested in this study) but even if this provides adequate recall will suffer the same problem of elevated false hits in its native form without additional filtering. In addition, Taxonstand currently undertakes near matching only on species epithets and therefore will not return any result in the presence of a misspelled genus name.

FishBase [47] presents an approach based on substring matching, previously documented in [48]. If a binomial name known to the system is entered no near match is attempted, otherwise the system presents names with a match on genus but not on species, matches on species but not on genus, then any binomial names where both the first and last two letters of genus and species epithet are the same. While in the majority of cases this will return lists of candidate names which most likely include the desired target, in some cases such lists will be quite long, for example in the case of genera with a large number of species, or commonly used species epithets, and no attempt is made to filter the more from the less similar names offered.

One might also consider whether standard (not specifically taxonomic) available approaches might also be suitable, for example the open source java text search engine Lucene [49] from Apache is available for many platforms and incorporates a near match facility ‘FuzzyQuery’ based on Damerau-Levenshtein (or optionally: Levenshtein) distance up to a maximum of ED 2, plus an *n*-gram indexing technique is incorporated in its ‘SpellChecker’ component. While Lucene may therefore perform well for some or many misspelled names, it will miss the more distant matches and, as in the cases above, without additional dedicated filtering will almost certainly return an elevated proportion of false positives.

### Comparison with previous findings in the taxonomic and general information retrieval domains

As noted in the introduction, Damerau's original (1964) premise that ‘over 80%’ of misspellings in plain text were attributable to a single character insertion, deletion, substitution or transposition has been broadly confirmed in this study (79.7% at DLD 1 for species binomials, 89.7% for genera) in the context of taxonomic names. Dalcin [12] reported 63.1% LD 1 errors at species level only in his study, which does not include single character transposition errors, and 93.4% recall by LD 2. From his Figure 29 he also reported 84% recall for bigrams and 60% for trigrams on species level names, apparently using a fixed threshold of 0.75 per name component (as a summed threshold of 1.5 for binomials); recall values in this study are somewhat higher at equivalent similarity thresholds. His Soundex recall rate was 74% for species, again with degraded levels of precision, as compared with around 85% in the present study. In his study, the only algorithm on test which was capable of 100% recall was the LD edit distance using a threshold of ED 4, although arguably the same result could have been achieved at some point for bigrams and trigrams by lowering the thresholds until all true hits were returned.

In terms of strategic approaches, the result shaping option employed in Taxamatch extends the manually designated (semi-) equivalent process potentially available in the GRIN Taxonomic Nomenclature Checker, in which a user would most likely search initially at a moderate edit distance (the value of LD 2 is presented as a default) and then perhaps manually increase the threshold recursively if no hits were returned. Taxamatch automates this step and in practice only requires to perform the search once since all results are generated in a single pass with the more distant hits simply being masked in the presence of closer ones using the default result shaping option, or exposed automatically where needed.

The strategy of a using a 2-stage filter, i.e. a computationally cheap ‘coarse’ filter as a prelude to a more expensive but accurate comparison, as employed in Taxamatch at both genus and species levels, is a well known optimization technique to aid algorithm efficiency in other domains. In analogous areas of information such as the retrieval of census data [26] and medical name matching (e.g. [25]) the term ‘blocking’ has been introduced, referring initially to geographic census blocks, used as a pre-filter device so that input names might be compared only with other names in the same ‘block’ rather than against all names. By extension, blocking can be also be envisaged based upon other extrinsic characteristics of the data (for example in personal name matching, requiring a match on gender, year of birth, data source, etc.) or intrinsic characters (such as a match on initial letter of both input and target terms, which as we have already seen is a feature of Soundex and some other related phonetic algorithms). Blocking could also be carried out (and tests repeated as parallel or sequential passes) using multiple criteria if no single one is considered be 100% reliable. One difference between such an approach and Taxamatch is that with classic blocking techniques, the assignment of target terms to blocks is static and can be carried out in advance; however with the Taxamatch approach all names must be available for initial consideration since the ‘blocking’/pre-filter approach is dynamic and depends upon a range of characteristics of the input name which cannot be determined in advance.

A related approach known as the sorted neighbourhood method [2] involves sorting the target data using some criterion (let us say alphabetic order in the first instance), determining the closest match point for the input term, and then testing target terms either side of the nearest match within a pre-defined window (for example 50 names below and 50 above the match point); once again, this might be repeated using a range different sort criteria on the basis that if the criteria are well chosen, using at least one of them the input term and the desired target should sort close together. Such an approach has been described by Müller et al. [50] in the area of taxonomic content matching using not only the name but also other extrinsic criteria such as collection, accession number and more, in order to reduce numbers of names for comparison to a manageable level, although in this case no test results were provided for evaluation of the degree of success or failure of this technique.

A final area to be briefly mentioned is that in which Taxamatch, or a similar near match function, provides one component of a more complete workflow, for example not only resolving a misspelled to its correctly spelled equivalent but providing additional information on its taxonomic status (plus resolving synonyms to current names), taxonomic placement and more. Such a facility is in fact provided in the Taxamatch-enabled IRMNG system already cited utilizing additional IRMNG content to just the taxonomic names, and has more recently been enabled (again using Taxamatch) in other online systems such as OBIS, the Ocean Biogeographic Information System [51], WoRMS, the World Register of Marine Species [52], PESI, the Pan-European Species Directories Infrastructure [53], the iPlant Taxonomic Name Resolution Service (TNRS) [54] and more, the latter system also being the subject of additional documentation in Boyle et al. [55]. At present the Taxamatch component of such systems is hard wired into the respective workflows but one could conceive of a further degree of modularization or decoupling (perhaps via web services) whereby the near matching and the subsequent further name resolution might occur at different locations or using different datasets, or where users could plug in a selection of different reference datasets to test against. A somewhat similar approach in this regard is already offered by the Global Names Resolver [56], again using a Taxamatch-derived near matching element, wherein a user-supplied set of names can be tested against either all or a subset of seven available reference datasets stored locally (for additional detail refer Table 5).

**Table 5:** Summary of current Taxamatch-enabled taxonomic data systems known to the author as at August 2013.

System name	Acronym	Year available	Content	Remarks	Taxamatch implementation
Interim Register of Marine and Nonmarine Genera [40]	IRMNG	2007	465,000 genus names and 1.67 million species at May 2013, all groups, extant and fossil	Emphasis on genus-level completeness at the present time. Incorporates the author's reference Taxamatch implementation.	Oracle PL/SQL – reference implementation [57]
SilverBiology SilverArchive [58]	-	-	Not known	Product development apparently in abeyance, but source code available	SilverBiology PHP [59]
University of Vienna Herbarium [60]	-	2009	Source databases currently include Vienna virtual herbarium, Catalogue of Life 2010 and 2011 editions and Fauna Europaea versions 1 and 2		PHP (custom)
Euro+Med PlantBase [61]	-	2009	Vascular plants of Europe and the Mediterranean region		PHP (custom)
World Register of Marine Species [52]	WoRMS	2010	381,000 species names, other ranks not stated, fairly complete for global marine species, the majority extant with a small number fossil	Includes several dozen component systems with individual identities e.g. World List of Porifera, Belgian Register of Marine Species, etc, refer http://www.marinespecies.org/about.php	Modified SilverBiology PHP
Pan-European Species Directories Infrastructure [53]	PESI	2010	An integration of Fauna Europaea for all European land and freshwater animals, Euro+Med PlantBase (refer own entry above), the European Register of Marine Species and the EU component of Index Fungorum		As per WoRMS
Atlas of Living Australia National Species Lists project [62]	ALA-NSL	2011	Integration of names, taxa and references in the Australian Faunal Directory (AFD), the Australian Plant Name Index (APNI) and the Australian Plant Census (APC)		Java [63]
The Global Names Index [36]	GNI	2011	17 million names (all ranks), all groups	No fuzzy search option offered, but uses Taxamatch as pre-processing to create ‘lexical groups’	Ruby [64]
Biodiversity Information Group IOZ [65]	-	2011			PHP (?)
The iPlant Taxonomic Name Resolution Service [54]	iPlant TNRS	2012	Choice of source databases for matching plant names: the Missouri Botanic Garden's Tropicos database, the Global Compositae Checklist and the United States Department of Agriculture PLANTS database		Extended SilverBiology PHP [66]
The Global Names Resolver [56]		2013	Checks input names for exact matches against up to 7 large reference sources concurrently including Catalogue of Life, ITIS, Index Fungorum, GBIF taxonomic backbone, IPNI, Encyclopedia of Life and Union	Fuzzy search appears to return a result from only a single resource at this time	As per Global Names Index

## Current Taxamatch Implementations Plus Source Code and Data Availability

Over the period since its initial construction and dissemination, Taxamatch has been incorporated into a number of significant regional and global biodiversity information systems as shown in Table 5. A copy of the present ‘reference’ Oracle PL/SQL implementation of Taxamatch is available from [57], with implementations in other languages including PHP+MySQL, Java and Ruby available from third parties as indicated under a range of licenses including Apache 2.0 ([57], [58]), Mozilla Public License 1.1 ([59]), BSD ([60]) and Gnu Lesser GPL ([61]). The version of IRMNG used for the testing (last update: 2013-01-11) is available for download from [57] and will shortly also be available via the Global Names Classification and List Repository (GNACLR) [62].

## Summary

This study demonstrates that a hybrid approach incorporating both a Modified Damerau-Levenshtein Distance algorithm and a phonetic algorithm customized to the characteristics of taxonomic names can detect close to 100% of errors in taxon scientific names, of multiple error types, and that good levels of both precision (rejection of false hits) and efficiency (algorithm performance) can be obtained via incorporation of appropriate rule-based filters at relevant points in the algorithm design. Efficiency is further enhanced by separate consideration of genus and species epithet portions, resulting in only a small number of epithets requiring to be tested once the initial set of near match genera has been obtained. The inclusion of a result shaping stage effectively automates the selection of relevant accept/reject thresholds on the basis of real-time results and requires no intervention from the user. Some residual problems with elevated levels of false hits in the case of genus names alone, in particular with shorter names, can be further assisted by the inclusion of a taxonomic or other filter in addition to a purely lexical component, where such additional information is available, or simply by use of a smaller, domain-specific reference dataset.

In tests using three sets of misspelled species names and four of genera alone, Taxamatch performance is shown to be superior in both recall and precision to other algorithms evaluated, which include Soundex and two custom phonetic algorithms, the *n*-gram variants bigrams and trigrams and either Levenshtein Distance, Damerau-Levenshtein Distance, or Modified Damerau-Levenshtein Distance used at fixed thresholds and without additional filtering. The three phonetic algorithms execute more rapidly but are deficient in recall of non-phonetic errors in particular, while the remaining (dynamic programming) algorithms show improved recall of most non-phonetic errors but are unacceptably slow in operation without additional substantial pre-filtering or blocking as introduced in Taxamatch. Taxamatch performance time per input name, at around 1.5 seconds per query, is considered reasonable in the present web-enabled system when searching against a reference database containing 465,000 genus and 1.67 million species, a value which might be improved further with additional optimization of either software or hardware environments, or by use of a faster version of Taxamatch described herein as ‘Taxamatch rapid’ for data-intensive tasks such as deduplication of very large datasets.

## Supporting Information

File S1 **Name pairs used in the present study for experimental algorithm comparisons (seven datasets), with results (as number of true/false hits) in each case.**
(XLS)

File S2 **The MDLD (modified Damerau-Levenshtein Distance) algorithm (Oracle PL/SQL implementation) as devised for this study, courtesy Barbara Boehmer (U.S.A.).**
(DOCX)

File S3 **A pseudocode representation of Taxamatch.**
(DOCX)
