# Supplementary material for: Taxamatch, an Algorithm for Near (‘Fuzzy’) Matching of Scientific Names in Taxonomic Databases
Source: PLoS One. 2014 Sep 23;9(9):e107510. doi: 10.1371/journal.pone.0107510 (PMC4172526; doi:10.1371/journal.pone.0107510)
Supplement: File S2 — The MDLD (modified Damerau-Levenshtein Distance) algorithm (Oracle PL/SQL implementation) as devised for this study, courtesy Barbara Boehmer (U.S.A.). (DOCX) [file pone.0107510.s002.docx]

Supplementary file S2. The MDLD (modified Damerau-Levenshtein Distance) algorithm (Oracle PL/SQL implementation) as devised for this study, courtesy Barbara Boehmer (U.S.A.).

(Supplement to Rees, Tony, 2014: Taxamatch, an Algorithm For Near (‘Fuzzy’) Matching of Scientific Names in Taxonomic Databases. PLOS One.)

-- Function: mdld

-- Purpose: Perform Modified Damerau-Levenshtein Distance test on two input strings, supporting block transpositions of multiple characters

-- Authors: Barbara Boehmer and Tony Rees (baboehme@hotmail.com, Tony.Rees@csiro.au)

-- Date created: March 2008

-- Inputs: string 1 as p_str1, string 2 as p_str2, numeric limit on length of transposed block to be searched for as p_block_limit

-- Outputs: computed edit distance between the input strings (0=identical on this measure, 1..n=increasing dissimilarity)

-- Remarks:

-- (1) Block limit must be 1 or greater. If set to 1, functions as standard Damerau-Levenshtein

-- Distance (DLD) test; for MDLD, setting block limit to a moderately low value (e.g. 3) will avoid excessive run times

-- (2) This is an extension of B. Boehmer’s original (2002) PL/SQL Levenshtein Distance function, available at

-- http://web.archive.org/web/20120526084237/http://www.merriampark.com/ldplsql.htm

-------------------------------------------------------------

CREATE OR REPLACE FUNCTION mdld

(p_str1 VARCHAR2 DEFAULT NULL,

p_str2 VARCHAR2 DEFAULT NULL,

p_block_limit NUMBER DEFAULT NULL)

RETURN NUMBER

AS

v_str1_length PLS_INTEGER := NVL (LENGTH (p_str1), 0);

v_str2_length PLS_INTEGER := NVL (LENGTH (p_str2), 0);

v_temp_str1 VARCHAR2 (32767);

v_temp_str2 VARCHAR2 (32767);

TYPE mytabtype IS TABLE OF NUMBER INDEX BY BINARY_INTEGER;

TYPE myarray IS TABLE OF mytabtype INDEX BY BINARY_INTEGER;

v_my_columns myarray;

v_empty_column mytabtype;

v_this_cost PLS_INTEGER := 0;

v_temp_block_length PLS_INTEGER;

BEGIN

IF p_str2 = p_str1 THEN

RETURN 0;

ELSIF v_str1_length = 0 OR v_str2_length = 0 THEN

RETURN GREATEST (v_str1_length, v_str2_length);

ELSIF v_str1_length = 1 AND v_str2_length = 1 AND p_str2 != p_str1 THEN

RETURN 1;

ELSE

v_temp_str1 := p_str1;

v_temp_str2 := p_str2;

-- first trim common initial characters

WHILE SUBSTR (v_temp_str1, 1, 1) = SUBSTR (v_temp_str2, 1, 1) LOOP

v_temp_str1 := SUBSTR (v_temp_str1, 2);

v_temp_str2 := SUBSTR (v_temp_str2, 2);

END LOOP;

-- then trim common trailing characters

WHILE SUBSTR (v_temp_str1, -1, 1) = SUBSTR (v_temp_str2, -1, 1) LOOP

v_temp_str1 := SUBSTR (v_temp_str1, 1, LENGTH (v_temp_str1) - 1);

v_temp_str2 := SUBSTR (v_temp_str2, 1, LENGTH (v_temp_str2) - 1);

END LOOP;

v_str1_length := NVL (LENGTH (v_temp_str1), 0);

v_str2_length := NVL (LENGTH (v_temp_str2), 0);

-- then calculate standard Levenshtein Distance

IF v_str1_length = 0 OR v_str2_length = 0 THEN

RETURN GREATEST (v_str1_length, v_str2_length);

ELSIF v_str1_length = 1 AND v_str2_length = 1 AND p_str2 != p_str1 THEN

RETURN 1;

ELSE

-- create columns

FOR s in 0 .. v_str1_length LOOP

v_my_columns (s) := v_empty_column;

END LOOP;

-- enter values in first (leftmost) column

FOR t in 0 .. v_str2_length LOOP

v_my_columns (0) (t) := t;

END LOOP;

-- populate remaining columns

FOR s in 1 .. v_str1_length LOOP

v_my_columns (s) (0) := s ;

-- populate each cell of one column:

FOR t in 1 .. v_str2_length LOOP

-- calculate cost

IF SUBSTR (v_temp_str1, s, 1) = SUBSTR (v_temp_str2, t, 1) THEN

v_this_cost := 0;

ELSE

v_this_cost := 1;

END IF;

-- extension to cover multiple single, double, triple, etc character transpositions

-- that includes calculation of original Levenshtein distance when no transposition found

v_temp_block_length := LEAST ( (v_str1_length / 2), (v_str2_length / 2), NVL (p_block_limit, 1));

WHILE v_temp_block_length >= 1 LOOP

IF s >= (v_temp_block_length * 2) AND

t >= (v_temp_block_length * 2) AND

SUBSTR (v_temp_str1, s - ( (v_temp_block_length * 2) - 1), v_temp_block_length) =

SUBSTR (v_temp_str2, t - (v_temp_block_length - 1), v_temp_block_length) AND

SUBSTR (v_temp_str1, s - (v_temp_block_length - 1), v_temp_block_length) =

SUBSTR (v_temp_str2, t - ( (v_temp_block_length * 2) - 1), v_temp_block_length) THEN

-- transposition found

v_my_columns (s) (t) := LEAST

(v_my_columns (s) (t - 1) + 1,

v_my_columns (s - 1) (t) + 1,

(v_my_columns (s - (v_temp_block_length * 2)) (t - (v_temp_block_length * 2))

+ v_this_cost + (v_temp_block_length - 1)));

v_temp_block_length := 0;

ELSIF v_temp_block_length = 1 THEN

-- no transposition

v_my_columns (s) (t) := LEAST (v_my_columns (s) (t - 1) + 1,

v_my_columns (s - 1) (t) + 1,

v_my_columns (s - 1) (t - 1) + v_this_cost);

END IF;

v_temp_block_length := v_temp_block_length - 1;

END LOOP;

END LOOP;

END LOOP;

END IF;

RETURN v_my_columns (v_str1_length) (v_str2_length);

END IF;

END mdld;
