# Supplementary material for: Taxamatch, an Algorithm for Near (‘Fuzzy’) Matching of Scientific Names in Taxonomic Databases
Source: PLoS One. 2014 Sep 23;9(9):e107510. doi: 10.1371/journal.pone.0107510 (PMC4172526; doi:10.1371/journal.pone.0107510)
Supplement: File S3 — A pseudocode representation of Taxamatch. (DOCX) [file pone.0107510.s003.docx]

Supplementary file S2. A pseudocode representation of Taxamatch.

(Supplement to Rees, Tony, 2014: Taxamatch, an Algorithm For Near (‘Fuzzy’) Matching of Scientific Names in Taxonomic Databases. PLOS One.)

function taxamatch(input_genus : String, input_species_epithet : String, input_authority : String, shaping_on : Boolean):

Requirements:

Genera //set of target genus names for testing, plus a unique identifier as needed. ‘Near match’ phonetically transformed version of the genus name and genus length would normally be pre-computed and cached in the database to avoid computation overhead at run time.

Species //set of target species names for testing, each comprising genus (as included in ‘Genera’) + species epithet + authority (as available), plus a unique identifier as needed, and foreign key to relevant genus identifier as applicable. ‘Near match’ phonetically transformed version of the species epithet and epithet length would normally be pre-computed and cached in the database to avoid computation overhead at run time.

near_match(str, word_type) //function to produce ‘Rees 2007 near match’ version of an input string ‘str’; if word_type = ‘genus_only’, does not perform supplementary operations on the end of the word; if word_type = ‘species_only’, does so; if not supplied, treats first word as a genus, and second/subsequent ones as epithets

mdld(string_1, string_2, block_limit) //function to compute MDLD (modified Damerau-Levenshtein Distance) between ‘string 1’ and ‘string 2’, including checking for transposed character blocks of length up to ‘block limit’ (refer note 1)

display_genus_details(species, input_authority) //function to display (or add to a list, e.g. for writing to a database table or report) a species name as a near match, with associated detail as required; optionally to also return a computed similarity value between the input authority and the authority of that species, e.g. using weighted n-gram measure or other. Incorporates ranking as desired: e.g. phonetic and ED1 matches as ‘nearest matches’, others as ‘other near matches’

display_species_details(species, input_authority) //function to display (or add to a list, e.g. for writing to a database table or report) a species name as a near match, with associated detail as required; optionally to also return a computed similarity value between the input authority and the authority of that species, e.g. using weighted n-gram measure or other. Incorporates ranking as desired: e.g. phonetic and ED1 matches as ‘nearest matches’, others as ‘other near matches’

Accepted_Species = Φ //intermediate set of ‘near match’ species names as generated during Taxamatch operation, each comprising a unique identifier per species, combined edit distance on both genus + species epithet cf. input species name, plus (as desired) genus + species epithet + authority (refer note 2, 4)

Displayed_Species = Φ //set of ‘near match’ species names as final Taxamatch output, each comprising a unique identifier per species plus (as desired) genus + species epithet + authority (refer note 2, 3, 4)

function satifies_genus_heuristic_1(genus): //selects the main set of genera for testing

if absolute(length(name_of(genus)) - length(input_genus)) < 3:

return True if

- length of (shortest) genus name < 5 and either the first 2 or last 2 characters match

- length of (shortest) genus name = 5 and either the first 2 or last 3 characters match

- length of (shortest) genus name >= 6 and either the first 3 or last 3 characters match

else:

return False

function satisfies_genus_heuristic_2(genus, species): //selects subset of genera that have the same ‘near match’ species

near_input_species_name = near_match(input_species, ‘species_only’)

near_species_name = near_match(name_of(species), ‘species_only’) //cached in database

if near_species_name = near_input_species_name and absolute(length(name_of(genus)) - length(input_genus)) < 4:

return True

else:

return False

function satisfies_genus_heuristic_3(genus): //selects genera that are a phonetic match

near_input_genus_name = near_match(input_genus, ‘genus_only’)

near_genus_name = near_match(name_of(genus), ‘genus_only’) //cached in database

if near_genus_name = near_input_genus_name:

return True

else:

return False

function satisfies_genus_name_prefilter(genus):

near_genus_name = near_match(name_of(genus), ‘genus_only’) //cached in database

if satisfies_genus_heuristic_1(genus)

or satisfies_genus_heuristic_2(genus, species)

or satisfies_genus_heuristic_3(genus):

return True

else:

return False

function satisfies_genus_name_postfilter(genus, edit_distance_genus):

if edit_distance_genus > 4:

return False

if near_match(input_genus, ‘genus_only’) = near_match(name_of(genus), ‘genus_only’):

return True

//require minimum 50% ‘good’ characters

if shortest length of {name_of(genus), input_genus} < 2 * edit_distance_genus:

return False

//first character must match for edit_distance 2+

if edit_distance_genus < 2 or first characters match:

return True

else:

return False

function satisfies_species_name_prefilter(species_epithet):

if absolute(length(name_of(species_epithet)) - length(input_species_epithet)) < 5:

return True

else:

return False

function satisfies_species_name_postfilter(species_epithet, edit_distance_species):

if edit_distance_species > 4:

return False

if near_match(input_species_epithet, ‘species_only’) = near_match(name_of(species_epithet), ‘species_only’): //cached in database

return True

//require minimum 50% ‘good’ characters

if shortest length of {name_of(species_epithet), input_species_epithet} < 2 * edit_distance_species:

return False

//first character must match for edit_distance 2 and 3, first 3 characters for edit distance 4

if edit_distance_species < 2

or edit_distance_species < 4 and first characters match

or edit_distance_species = 4 and first three characters match:

return True

else:

return False

//main Taxamatch algorithm is here

//select candidate genera for testing i.e. genus pre-filter

for all genus in Genera where satisfies_genus_name_prefilter(genus):

//do the MDLD comparison

edit_distance_genus = mdld(name_of(genus), input_genus, 2) //limit MDLD action with genera searched to max. 2-character transposed blocks, to avoid excessive run time

//add the genus post-filter

if satisfies_genus_name_postfilter(genus, edit_distance_genus):

//add genus result shaping if required (Taxamatch normal mode) or skip this step if requested (‘no shaping’ mode)

if shaping_on = false:

for all genus in Accepted_Genera

add genus to Displayed_Genera

if shaping_on = true: //genus result shaping: exact matches, phonetic matches and edit distance 1 always displayed, display of other edit distances conditional on no closer hits found

if satisfies_genus_heuristic_3(genus) = true: //selects genera that are a phonetic match

is_phonetic_match = true

for all genus in Accepted_Genera

where is_phonetic_match = true:

add genus to Displayed_Genera

for output_edit_distance from 0 to 1:

for all genus in Accepted_Genera

where edit_distance_combined = output_edit_distance and is_phonetic_match = false:

add genus to Displayed_Genera

if no genera displayed:

for output_edit_distance = 2:

for all genus in Accepted_Genera

where edit_distance_combined = output_edit_distance and is_phonetic_match = false:

add genus to Displayed_Genera

// proceed to species test if epithet is supplied

if input_species_epithet is not null:

//select candidate species for testing i.e. species pre-filter

for all species in Species

where genus_of(species) = genus

and satisfies_species_name_prefilter(species):

//do the MDLD comparison

edit_distance_species = mdld(name_of(species_epithet), input_species_epithet, 4) //can afford to search for species with max. 4-character transposed blocks, as generally substantially fewer than required genus tests

edit_distance_combined = edit_distance_genus + edit_distance_species

//add the species post-filter

if satifies_species_name_postfilter(species, edit_distance_species):

add species, edit_distance_combined to Accepted_Species

//add species result shaping if required (Taxamatch normal mode) or skip this step if requested (‘no shaping’ mode)

if shaping_on = false:

for all species in Accepted_Species

add species to Displayed_Species

if shaping_on = true: //species result shaping: exact matches, phonetic matches and edit distance 1, 2 always displayed, display of other edit distances conditional on no closer hits found

if shaping_on = true:

near_input_species_name = near_match(input_species, ‘species_only’)

near_species_name = near_match(name_of(species), ‘species_only’) //cached in database

if near_species_name = near_input_species_name

is_phonetic_match = true

for all species in Accepted_Species

where is_phonetic_match = true:

add species to Displayed_Species

for output_edit_distance from 0 to 2:

for all species in Accepted_Species

where edit_distance_combined = output_edit_distance and is_phonetic_match = false:

add species to Displayed_Species

if no species displayed:

for output_edit_distance = 3:

for all species in Accepted_Species

where edit_distance_combined = output_edit_distance and is_phonetic_match = false:

add species to Displayed_Species

if no species displayed:

for output_edit_distance = 4:

for all species in Accepted_Species

where edit_distance_combined = output_edit_distance and is_phonetic_match = false:

add species to Displayed_Species

if shaping_on = false:

for all species in Accepted_Species

add species to Displayed_Species

//result presentation, can include authority similarity testing as desired (refer note 4)

for all genus in Displayed_Genera

if input_species_epithet is null: //authority supplied (if any) belongs to genus

display_genus_details(genus, input_authority)

else:

display_genus_details(genus)

if input_species_epithet is not null:

for all species in Displayed_Species

display_species_details(species, input_authority)

Notes:

(1) Setting ‘block limit’ to the minimum value required in any particular stage avoids excessive run time

(2) The genus, species epithet and authority for each item in ‘Accepted_Species’ and ‘Displayed_Species’ need not be included in those sets provided that a unique species identifier is available, since they can be retrieved later via a separate call or calls to the relevant data table(s) (e.g. within the function ‘display_species_details’), however since at least the first two items have already been retrieved for testing, there may be performance advantages in doing so

(3) The set ‘Displayed_Species’ is included to illustrate the principle of the algorithm, but in practice could be replaced by individual calls to ‘display_species_details’ as a result of testing the values in ‘Accepted_Species’ at each point

(4) Authority testing is used here for information to user only, but could also be utilized as a contribution to ranking if desired, e.g. display closest authority matches first.
